# Osteopontin in the host response to *Leishmania amazonensis*

**DOI:** 10.1186/s12866-019-1404-z

**Published:** 2019-02-08

**Authors:** Emilie Giraud, Eline Rouault, Laurence Fiette, Jean-Hervé Colle, Despoina Smirlis, Evie Melanitou

**Affiliations:** 1 Immunophysiology and Parasitism Laboratory and Department of Parasites and Insect Vectors, Institut Pasteur, 28 rue du Dr Roux, 75724 Paris Cedex 15, France; 2 Human Histopathology and animal models Laboratory, Institut Pasteur, 28 rue du Dr Roux, 75724 Paris Cedex 15, France; 3 Nuclear Magnetic Resonance of Biomolecules unit, Institut Pasteur, 28 rue du Dr Roux, 75724 Paris Cedex 15, France; 4 Molecular Parasitology Laboratory, Microbiology Department, Hellenic Pasteur Institute, 127 Bas. Sofias Avenue, 11521 Athens, Greece; 5 Present address: Insect-Virus Interactions Laboratory / CNRS UMR2000, Institut Pasteur, 28 rue du Dr Roux, 75724 Paris Cedex 15, France; 6 Present address : GENOSAFE Laboratories, 1 rue de l’Internationale, Evry, 91000 France; 7 Present address: Institut Mutualiste Montsouris Research, Paris, France

**Keywords:** Osteopontin, Macrophage, Inflammation, Inflammasome, Pyroptosis, Parasites, *Leishmania amazonensis*, C57BL/6 mice

## Abstract

**Background:**

*Leishmania (L.) spp* are intracellular eukaryotic parasites responsible for cutaneous or visceral leishmaniasis, replicating predominantly in macrophages (MF). In C57BL/6 mice virulence with *L. amazonensis* has been associated with inhibition of Th1 immune responses and an uncontrolled lesion development, whereas DBA/2 mice control any lesion. Parasitic clearance by the MFs requires the activation of proper immune responses. One of the immune related genes expressed in immune cells including MF, codes for osteopontin (OPN). OPN is a secreted glycoprotein, acting as an immune regulator. Its implication in promoting Th1 immunity in response to infectious microorganisms and its known protective effect against viral and bacterial infections via activation of the immune response, render OPN a molecule of interest in the study of the host response to *L. amazonensis*.

**Results:**

We examined the host response to *L. amazonensis* of *opn* mutant and wild type C57BL/6 mice. Bone marrow derived MFs were infected with the parasites in vitro*,* and *opn* mutant and wild type mice were inoculated in vivo by intradermal injection in the ears. The DBA/2 strain known to control *L. amazonensis* infection was also used for comparison. Our data indicate that the parasites increased *opn* gene expression and OPN protein while parasitic proliferation was contained in the presence of OPN. In the presence of parasites the expression of inflammation-related transcripts was inhibited. Interleukin-1-beta (IL-1β), and transcripts of the NLR–family (NLRC4, NLRP3) were down regulated after *L. amazonensis* infection. In the absence of OPN, the inhibition by the parasites of IL-1β transcripts was less efficient and a pyroptosis-like cell phenotype was detected in vitro, suggesting a central role of OPN in the host-response to *L. amazonensis*. Similarly, in vivo*,* in the absence of OPN, while the clinical inflammatory phenotype is more severe, an increase of these transcripts was observed.

**Conclusions:**

*L. amazonensis* infection induces *opn* gene expression and protein, which in turn participates in shaping the host response to the parasites, seemingly by decreasing the activation of inflammation. OPN, further evaluated as a target for Leishmaniasis control represents an additional interest in improving vaccination strategies against the parasites.

**Electronic supplementary material:**

The online version of this article (10.1186/s12866-019-1404-z) contains supplementary material, which is available to authorized users.

## Background

Parasites of the *Leishmania* (*L*.) genus are the causative agents of leishmaniasis in humans, a disease that affects more than 12 million people worldwide causing significant morbidity and mortality [1]. *Leishmania* parasites affect a variety of organs and tissues depending on the species, causing characteristic lesions in skin, mucosal surfaces and visceral organs. In particular, *L. amazonensis* is the causative agent of the human cutaneous form of the disease, frequently observed in the developing world [2]. *Leishmania* is transmitted at the metacyclic promastigote stage by the bite of infected phlebotomine sandflies of the genus *Phlebotomus* in the Old World and of the genus *Lutzomyia* in the New World. The primary hosts are mammals including rodents. During blood feeding from an infected host the sandfly ingests amastigote-infected cells. Amastigotes differentiate into the procyclic *Leishmania* promastigotes in the sandfly midgut [3, 4]. This is the replicative form of the parasite in the insect host. Then, this stage is marked by the arrest of replication and subsequent migration of the parasites to the insect proboscides whereas the metacyclogenesis takes place resulting to the differentiated infective form: the metacyclic promastigotes [5, 6]. The latter, once delivered into the mammal dermis during the blood feeding bite of the female insect, differentiate as amastigotes and are mainly found within the resident dermal macrophages (MF) [7] but also in dendritic cells (DC) [8].

The mechanisms by which *Leishmania* parasites persist and proliferate in these phagocytic cells are not completely understood. Parasitic burden seems to be inoffensive for the host cell and *Leishmania* proliferation is not prevented [8]. In particular macrophages while they represent the major effector cells for eliminating infection, they also are the primary resident cells for these parasites. Parasitic clearance by the macrophages requires the activation of proper immune responses. The host’s immune response may lead to resistance or susceptibility to the infection, while in mouse strains the response to *Leishmania spp* is dependent upon the genetic background.

In most mouse strains, subcutaneous inoculation of *L. amazonensis* leads to chronic infections with non-healing lesions similar to the ones observed with *L. major* in BALB/c mice [9]. Interestingly virulence of *L. amazonensis* in C57BL/6 mice has been associated with inhibition of CD4^+^ Th1 responses rather than promotion of Th2 innate immune response [10]. In contrast to the C57BL/6 mice, DBA/2 mice show sustained infection after *L. amazonensis* inoculation, compatible with a resistance phenotype to these parasites [9, 11].

One of the immune related genes expressed in MF, DC and T cells encodes for osteopontin (OPN), a secreted RGD-containing glycoprotein. It is an acidic phosphoprotein called also *Eta*-1 (Early T lymphocyte activation 1) or *spp1* (secreted phosphoprotein 1). Its pleiotropic effects include cell adhesion properties, cell signalling, migration and attachment [12]. Functions in bone remodelling, tumour metastasis and host defence also have been described [13, 14]. Osteopontin was originally identified as highly expressed by activated T cells [15, 16] and is considered a key cytokine that sets the stage for efficient type 1 immune responses through differential regulation of IL-12 and IL-10 cytokine expression in macrophages [17]. Mice deficient in *opn* gene expression have severely impaired type 1 immunity to viral and bacterial infections [17]. IL-12 and IFN-γ production is diminished in the *opn* mutant mice, and IL-10 production is increased. OPN also plays a major role in autoimmune diseases, in particular during remission/relapse in Multiple Sclerosis (MS) in human [18] and it was found to be up-regulated in the pancreas draining lymph nodes at the early stages of type 1 diabetes in the NOD mouse model [19].

In this work, we assessed the implication of OPN in the host response to *Leishmania amazonensis* parasites. Its implication in promoting Th1 immunity in response to infectious microorganisms and its expression in immune cells including MF [20, 21], makes it a good candidate possibly involved in the subversion of the host cells by *Leishmania*. Moreover the protective effect of OPN, against viruses (herpes simplex virus-type 1, KOS strain) [17] and bacterial infections (*Listeria monocytogenes*) [22] via activation of the immune response, suggests a potential role as a therapeutic target against cutaneous leishmaniasis. Over-expression of *opn* was shown in affected tissues in autoimmune conditions such as MS, rheumatoid arthritis and Crohn’s disease patients [23–25]. Interplay of OPN with the immune system, in infection and autoimmunity, indicates that this molecule may play a role in the infection-related environmental component of autoimmune diseases aetiology, as postulated by the “Old friends’ hypothesis” [26].

Intracellular parasites of the *Leishmania* species employ strategies preventing cell death and favouring sustained adaptation of the host cell during parasite replication. In particular, cell death may modulate inflammatory responses and influence the immune system; while on the contrary survival of the immune cells hosting *Leishmania* parasites may have significant impact on mechanisms which when understood may reveal novel therapeutic approaches.

We evaluated the implication of OPN in the host response to *L. amazonensis* in vitro, in bone marrow derived MF (BMFs) and in vivo, in the presence or absence of OPN protein. In the absence of OPN, a pyroptosis-like phenotype was observed in the BMFs in vitro, which incited to address the levels of the inflammasome-related transcripts in response to parasites in relation with *opn* gene expression. Our data demonstrate that the parasites induce the production of the osteopontin in the BMFs in vitro and on the sites of inoculation in vivo while they inhibited the pro-inflammatory transcripts. In the absence of OPN this inhibition was moderated in the BMFs as well as in vivo suggesting that this molecule plays a role in shaping the host response to the parasites.

## Results

### OPN restricts parasitic proliferation in vitro

**Fig. 1:**
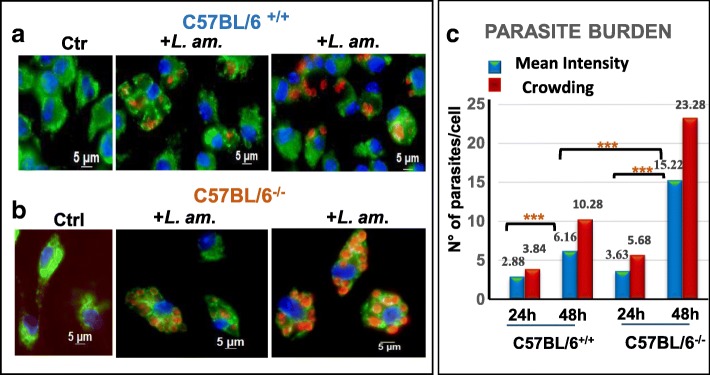
*L. amazonensis* proliferation in BMFs in the presence or absence of osteopontin. *L. amazonensis* amastigotes at a ratio 4:1, were added or not (Ctrl) to (**a**) C57BL/6^+/+^ or (**b**) *opn* mutant (C57BL/6^−/−^), representative images of BMFs populations. Twenty four and 48 h later, each BMFs population was analysed by immunostaining. Nuclei were stained with Hoechst (*blue*), vacuoles with Lysosome-associated membrane protein Lamp-1 Ab and FITC-labelled conjugate (*green***)** and amastigotes with 2A3-A26 Ab and Texas Red-conjugate (*red*). All images are in phase-contrast optical microscopy. **c**. Parasite Intensity: Mean intensities and intracellular parasite crowding of *L. amazonensis* amastigotes per infected cell was monitored by manual analysis of immunofluorescence image captures (AxioVision) of at least 2 different experiments (≥ 40 cells evaluated per condition). Statistical analyses were performed using the QP3.0 program designed for Quantitative parasitology as described in the Methods section. Mean intensities were compared by the Bootstrap test and 2-sided bootstrap *p*-values are given as follows: WT vs KO at 24 h, NS; WT vs KO 48 h, *P* = 0.0001; WT 24 h vs 48 h and KO 24 h vs 48 h, *P* = 0.00001. Mean crowding across samples was significant with non-overlapping confidence limits (Cl) at 97.5% and a *p*-value< 0.0595%, (95% Cl). Statistics are analytically presented on Additional file 2: Table S1A and B

To gain a first insight into a potential role of OPN during *L. amazonensis* infection, we evaluated the replication of the parasites in BMF from mice lacking the *opn* gene. We used BMF isolated from C57BL/6 wild type (C57BL/6^+/+^) and the *opn* mutant mice, (C57BL/6^−/−^). After differentiation to macrophages, more than 95% of these cells were CD11b high, F4/80 high, Cd115 high and CD11c negative (Additional file 1: Figure S1). In BMFs after 24 h and 48 h post infection in the absence of *opn*, parasites showed increased intracellular proliferation by immunostaining (Fig. 1a versus b). Evaluation of parasite intensity confirmed increased parasite numbers in the *opn* mutant cells (Fig. 1c), in particular, mean intensity and parasite crowding differences between the *opn* WT and KO macrophages were more pronounced at 48 h *p.i*. (*P* = 0.0075, CI 95% and *P* < 0.05, CI 95% respectively), (Fig. 1c) and Additional file 2: Table S1A). These data were confirmed by RT-qPCR (data not shown). Therefore in the absence of *opn,* intracellular proliferation of parasites is higher than in the wild type cells at 48 h *p.i.*, demonstrating an OPN-related parasitic restriction effect.

The population prevalence (proportion of the infected host cells among all the host cells examined) between the *opn* wild type and mutant BMFs, was higher in the *opn* KO samples (*p* = 0.002, CI 95%) at 24 h *p.i.* suggesting an OPN-related restriction of the infection rates (Additional file 2: Table S1B).

### *Leishmania amazonensis* parasites induce *opn* gene expression and OPN protein in BMFs

The ability of parasites to survive within the macrophages and the resistance of the host cells to their leishmanicidal activity, together with the restrictive effect of OPN on parasite proliferation, described above, indicate an OPN-related host defence response to parasites.

**Fig. 2:**
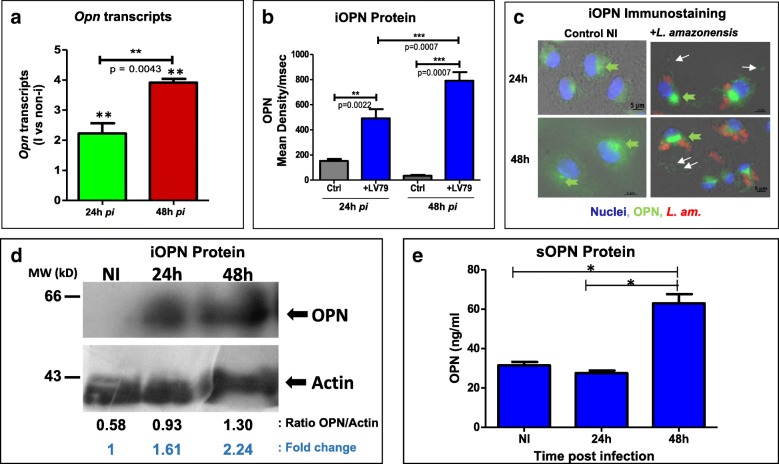
*L. amazonensis* parasites stimulate OPN in BMFs. **a**. *Opn* transcript concentrations were evaluated by real-time quantitative PCR. Relative quantity of *opn* transcripts is calculated vs the non-infected (NI) values. At 24 h *p.i.* NI vs I, *P* = 0.0022; at 48 h *p.i.* NI vs I, *P* = 0.005. **b**. Mean Density of OPN protein FITC staining with anti-OPN antibody as described in (**c**). > 10 cells were evaluated per sample. *Blue*: infected with LV79 amastigotes and *gray*: non-infected control values. **c**. Immunostaining of BMFs isolated from C57BL/6 wild type control (NI, Non-Infected) and infected with *L.amazonensis* amastigotes, at 24 h and 48 h *p.i*. OPN was labelled with anti-OPN antibodies and revealed with donkey anti-goat FITC (*green*). The mAb 2A3–26-biotinylated was used for the parasites and revealed with streptavidin conjugated to Texas Red (*red*). Nuclei are in *blue* (Hoechst). Green arrows indicate the intracellular OPN, seemingly concentrated in the ER. White arrows indicate secretory granules and sOPN. **d**. Detection and quantification of intracellular (iOPN) by Western blot analysis of BMF cell extracts from C57Bl/6 wild type mice, non-infected (NI) and infected with the parasites for 24 h and 48 h. 15 μg of total protein extracts were loaded per lane; Molecular weight markers positions are shown on the left of the figure. Below are the OPN/Actin ratio and the fold changes are noted in comparison with the non-infected OPN stain. **e**. Quantification of the secreted form of OPN (sOPN) in the BMF media (100 μl) at various times post infection by an ELISA assay

We evaluated the *opn* gene expression in the BMFs following infection with *L. amazonensis* parasites*.* Relative quantity of *opn* transcripts was assessed by RT-qPCR, at 24 h and 48 h *p.i.* (Fig. 2a) and the presence of intracellular iOPN protein was evaluated in cells by immunostaining with anti-OPN antibodies (Fig. 2b and c) and by Western blot analysis (Fig. 2d). Quantification of *Opn* transcripts showed a time-dependent increase in infected BMFs from wild type C57BL/6 mice, (*p* = 0.0043, Fig. 2a). Comparison of the *opn* transcripts between non-infected and infected cells showed a 2.2 and threefold increase at 24 h *p.i*. (*p* = 0.0022) and 48 h *p.i*. (*p* = 0.005) respectively (Fig. 2a). A constitutive expression of OPN in macrophages was identified by immunostaining as non-infected BMFs also expressed OPN (Fig. 2c). These experiments revealed: **i**) an increase of the amount of OPN protein between infected (mean density: 492/msec, at 24 h *p.i*. and 791/msec, at 48 h p.i.) and non-infected cells (mean density: 157/msec, at 24 h *p.i*. and 35/msec, at 48 h *p.i*.) (Fig. 2b) and ii) a variation of stain intensity between the secreted and intracellular forms of the protein (Fig. 2c, pointed by the arrows). Western blot analysis of the infected with *L. amazonensis* axenic amastigotes BMF cell extracts, confirmed the increase of OPN protein in response to parasites observed by immunostaining (Fig. 2d). A time dependent OPN protein accumulation is observed reaching 2.24 fold the non-infected levels at 48 h *p.i*. (Fig. 2d). The absence of a clear signal in the non-infected samples possibly indicated the sensitivity of the nitrocellulose membranes to bind small quantities of OPN protein. However in other exposition time a faint band was clearly seen (data not shown).

Experiments attempting to target separately two *opn* transcripts by RT-qPCR, as it was previously reported in the MF [27] and pDC cells [28] corresponding to two OPN isoforms, failed to demonstrate the presence of alternative splicing, indicating that the presence of the parasites does not affect *opn* transcripts (Additional file 3: Figure S2). Therefore in our experiments, both the intracellular (iOPN) and full length forms of the protein (FLOPN) observed in the BMFs infected with the parasites (Fig. 2c), are not due to two different mRNA molecules but to alternative translation of the protein or to a delay of the secretory OPN form to exit the cells. Immunostaining detected possibly the secreted OPN (sOPN) inside the cells as seen by signals corresponding to the secretory granules but also at the extracellular space (Fig. 2c, white arrows). The intracellular fluorescent staining seems to indicate nascent OPN protein molecules in the endoplasmic reticulum (ER) (Fig. 2c, green arrows) and was also confirmed by Western blot analysis of the cell extracts (Fig. 2d). We evaluated the secreted OPN (sOPN) protein in the BMF media in response to parasites by an Elisa assay (Fig. 2e). Our data show that while an intracellular form of OPN is observed at 24 h *p.i*. (Fig. 2b-d) the secreted OPN protein increases in the cell media only after 48 h post infection (reaching 62.95 ng/ml vs 27.57 ng/ml at 24 h and 31.5 the control) (Fig. 2e).

The increase of *opn* transcripts in the presence of *Leishmania* parasites over time, reaching three fold the non-infected cell levels at 48 h (Fig. 2a), indicates that restriction of parasite proliferation in vitro is linked to the endogenous presence of OPN in the macrophages. Moreover evaluation of the CD44 gene expression, coding for an OPN receptor remained similar in both wild type and *opn* mutant mice (Additional file 4: Figure S3) suggesting a CD44 receptor independent effect of the OPN molecule.

The increased parasitic load detected in the absence of *opn* and the higher *opn* transcript levels and OPN protein observed after the addition of parasites, indicate that OPN confers protection to the host cell by containing the intracellular parasite number but not eliminating the invading microorganisms.

### OPN prevents a pyroptosis-like cell phenotype and shields macrophages against parasites

**Fig. 3:**
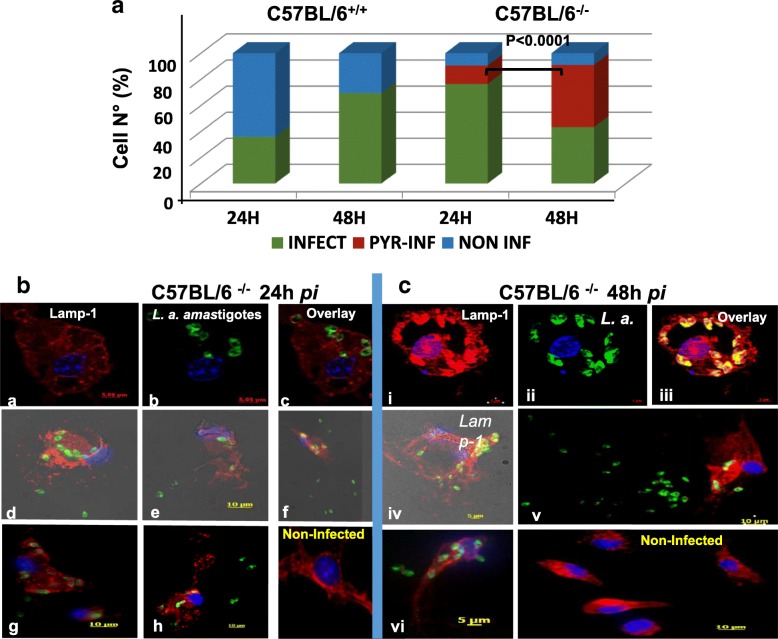
Distinct phenotypic traits of the *opn* mutant BMFs, in the presence of *L. amazonensis* parasites. **a**. Phenotypic evaluation of BMFs isolated from wild type (C57BL/6^+/+^) and *opn* mutant mice (C57BL/6^−/−^). While in the presence of *opn*, the pyroptosis-like phenotype is not observed, in its absence (C57BL/6^−/−^), 14.15 and 48% of the cells infected (INF) with the amastigotes are undergoing to a pyroptosis-like cell death (PYR) at 24 h and 48 h *p.i.* respectively (total cell N° counted at both time points: C57BL/6^+/+^: 416 and C57BL/6^−/−^ 385). Statistical analyses are performed by parametric Z-test, comparison of two proportions. **b**. Immunostaining of BMFs from C57BL/6.*spp1*^−/−^ mice infected with LV79 *L. amazonensis* amastigotes at a MOI: 4:1. Representative cell phenotypes of non-pyroptotic cells at 24 h *p.i*. (**a**-**c**) and at 48 h *p.i. (*i-iii) and cells undergoing to pyroptosis-like cell death are shown at 24 h *p.i*., (d-h) and at 48 h *p.i.*, (iv-vi) (phase contrast image capture). (*Lamp-1*, *LV79*, *Nuclei*)

With the aim to address the role of OPN in cell infectivity and survival in the presence of parasites, we evaluated by immunostaining the number of BMFs cells infected with the parasites (prevalence) at 24 h (Total N° of cells: WT: 206; KO: 318) and 48 h *p.i*.(Total N° of cells: WT:210; KO: 67), (Fig. 3a). These data suggested that in the *opn* mutant mice the proportion of infected cells was higher (92.5%) in comparison with the wild type macrophages (61%) at 48 h (Fig. 3a and Additional file 5: Table S2).

Strikingly parasite localization within cell compartments as seen by immunostaining showed that a fraction of macrophages from *opn* mutant mice, harbouring parasites, seemingly exhibited loss of cell integrity at 24 h *p.i.* and 48 h *p.i* (C57BL/6^−/−^, Fig. 3b and c, respectively). This phenomenon could relay to a pyroptosis-like phenotype [29] and was only observed to the cell cultures from *opn* deficient mice. However not all the *opn* mutant cells presented this phenotype (Fig. 3b, a-c versus d-h and 3C, i-iii versus iv-vi).

At 24 h *p.i.* 10.6% of the cells showed a pyroptosis-like phenotype, while at 48 h *p.i.* almost half of the cell population (48%) seemed to be pyroptotic-like (Fig. 3a). However while 90% of the *opn* mutant cells are infected, over 30% of the wild type cells expressing OPN remain parasite-free (Fig. 3a). Hence both cell phenotypes (pyroptotic-like and intact) are present in the absence of *opn* at 24 h and 48 h *p.i.* in the BMFs (Fig. 3a).

Overall these data indicate that not only parasite proliferation takes place but most likely higher infectivity also accounts for the increased numbers of parasites in infected cells from the *opn* deficient mice.

### *Leishmania amazonensis* inhibits the *IL-1β* transcripts in BMFs: Implication of OPN

**Fig. 4:**
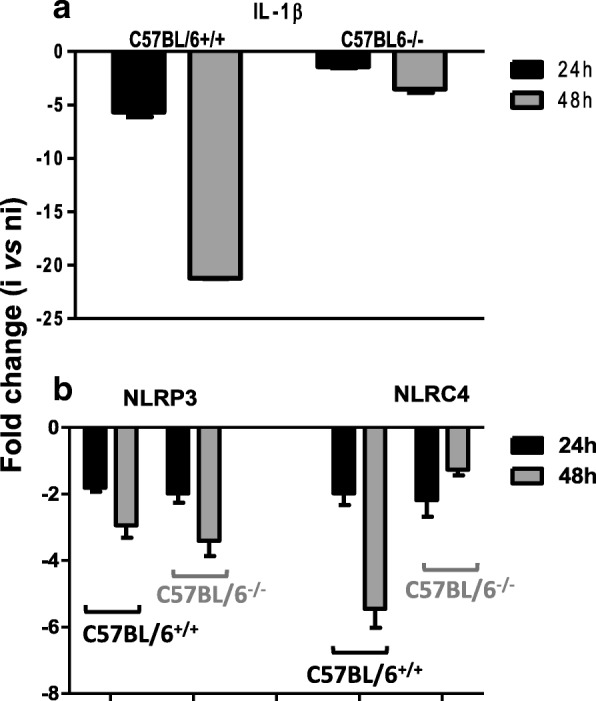
Host inflammasome and innate immune response to *L. amazonensis* parasites. **a**. Evaluation by RT-qPCR of IL-1β transcripts in T-RNA isolated from BMFs infected with *L. amazonensis* amastigotes of C57BL/6^+/+^ and *opn* mutant mice (C57BL/6^−/−^) at 24 h and 48 h post infection. **b**. NLRP3 and NLRC4 inflammasome transcripts. Data are stated as the fold changes of transcripts in infected (I) at 24 h and 48 h *p.i*., versus the control values of the non-infected (non-i) cells at each time point

The higher OPN levels observed after infection with the parasites and the pyroptosis-like phenotype observed in the BMFs in the absence of *opn* incited us to explore the expression levels of inflammasome and cell-death related genes known to be present in macrophages and monocytes. Moreover the anti-apoptotic role of OPN in various cell types previously reported [30] indicates that this molecule may confer protection to the host cell or be beneficial to the parasites. In this line we examined the expression patterns of genes coding for IL-1β and the NLRP3 and NLRC4 inflammasomes (NOD-like receptor (NLR) family) (Fig. 4). The NLRP3 inflammasome is known to regulate the release of the pro-inflammatory cytokine IL-1β in response to danger signals including pathogens through the activation of Casp1 [31–33].

In the wild type BMFs, while *casp-1* expression is increased by *L. amazonensis* amastigotes (Additional file 6: Figure S4), the expression of transcripts coding for NLRP3 and NLRC4 (NOD-like receptor (NLR) family) are over 2 fold inhibited by the presence of parasites (Fig. 4b). In particular*, IL-1β* transcripts are over 10 fold down-regulated in the wild type MFs (C57BL/6^+/+^) at 48 h *p.i*. in comparison with the non-infected cells (Fig. 4a).

Our data show that the transcripts of the major players of the inflammasome NLRP3 and NLRC4 are down-regulated by *L. amazonensis* parasites, despite an increase of the *casp*1 transcripts in the presence of OPN (Fig. 4b). Similarly, inhibition of these genes was observed in the *opn* mutant BMFs indicating a strong effect of the parasites on this inhibition. Interestingly while the *IL-1β* transcripts were highly down regulated by the presence of the parasites in the *opn* wild type cells, in the *opn* mutant BMFs this inhibition was highly moderated (Fig. 1a). Although transcript quantification is usually not sufficient per se to evaluate the quantities of the corresponding proteins, down regulation of the expression of these genes suggests that the proteins are absent or present at very low levels in the cell. These data are in agreement with an inhibition of the inflammasome transcripts by *L. amazonensis* parasites observed in the BMFs (Fig. 4). The expression of genes coding for cell death-related molecules in response to the parasites was evaluated (Additional file 6: Figure S4). Transcripts levels were assessed for CASP-3 known to be activated by numerous death signals and to be implicated in apoptosis [34], as well as genes coding for the NAIP5, NOD2 and ASC [35]. The ASC or PYCARD gene (apoptosis-associated speck-like, containing a caspase-recruitment domain) is part of the inflammasome multiprotein complex [36] and is expressed at lower levels in the *opn*^−/−^ mice (Additional file 6: Figure S4).

NOD2 and NAIP5 function as cytosolic sensors for the induction of apoptosis and regulate the inflammatory responses by innate recognition of microorganisms [37, 38]. NAIP5 interacts with NLRC4 proteins to trigger the inflammasome by activation of CASP-1 [39]. In our data, wild type BMFs sense parasites probably through the activation of *Casp-1*, and NAIP5 (Additional file 6: Figure S4), while in the absence of *opn* the expression of these genes is abolished or down-regulated, showing, in this case, the shutdown of parasite recognition signals in the BMFs. Noticeably, the up-regulation of the *casp-1* and *NAIP5* transcripts in the *opn*^+/+^ wild type BMFs (over 2 fold, Additional file 6: Figure S4) was not sufficient to trigger inflammatory cell death in the presence of the parasites (Fig. 3a).

Overall our data indicate that while parasite sensors are activated in the presence of *L. amazonensis* (transcripts *casp-1* and *NAIP5* on Additional file 6: Figure S4) the parasites inhibit the inflammasome transcripts, independently of the presence of *opn* (Fig. 4b). The strongest effect observed in the absence of OPN is the moderation of the inhibitory effect of the parasites on the *IL-1β* transcripts detected in the wild type macrophages (Fig. 4a). Taking in consideration the inflammatory-related phenotypic changes observed in the *opn* mutant mice (Fig. 3 and Additional file 7: Figure S5), these data seem to be in agreement with a host-protective effect of the OPN against *Leishmania* proliferation. In the absence of OPN, a balance is taking place, poised by the adaptive ability of the cells to the invading pathogen and the host cell-defence mechanisms (over 50% of the infected cells remain intact, Fig. 3a). The rate of pyroptotic-like cells may however increase if *opn* mutant cells are exposed longer than 48 h to the parasites.

### In vivo inoculation of *L. amazonensis* metacyclic promastigotes in the ear pinna of C57BL/6 wild type and *opn* mutant mice elaborates distinct phenotypic variations

**Fig. 5:**
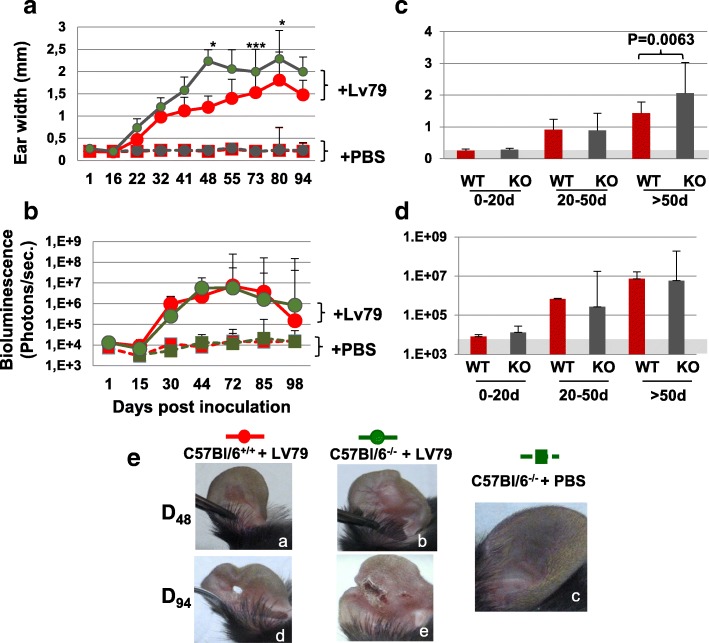
In vivo phenotypic evaluation and parasitic load. C57BL/6 wild type and *opn* mutant mice (C57BL/6^−/−^) mice were inoculated with 10^4^ *L. amazonensis* metacyclic promastigotes in the ear pinna*.* **a**. Longitudinal examination of clinical phenotype (ear width) at different time points post inoculation. Medians and SDs are indicated. Ear widths, *P* values: D48: *P* = 0.034; D73: *P* = 0.007; D80: *P* = 0.017. **b**. Monitoring of fluctuations of parasite load by bioluminescence. Signals are captured in the dermis of the ear pinnae. X axis represents days *p.i..* Results obtained from wild type mice (C57BL/6: *n* = 16 mice per group) are represented in red and from *opn* mutant mice (C57BL/6^−/−^, *n* = 23 mice per group) are represented in green. **c** and **d**. Time windows (days), after inoculation of 10^4^ metacyclic promastigotes of luciferase expressing *L. amazonensis* in the ear dermis of C57BL/6^+/+^ (WT) and *opn*^−/−^ (KO) mice. Animals were grouped for each time window as indicated on the X axis up to 98 days *p.i.* (WT: n = 16 mice, KO: n = 23 mice)*.* **c**. Comparison of clinical lesions at different time points post inoculation (ear widths) (Mann-Whitney test; *P* < 0.006). **d**. Comparison of parasitic load determined by bioluminescence signal quantification at different time points post inoculation (Medians and SD are indicated). **e**. Evolution of the ear lesions in representative mice at two different time points post-inoculation of parasites: at day 48 (**a**, **b**) and at day 94 (**d**, **e**); an image of a control non-inoculated ear is shown on (**c**)

To assess a role of OPN during *L. amazonensis* infection in vivo, C57BL/6 wild type and *opn* mutant mice were inoculated with 10^4^ metacyclic promastigotes, the infectious form of *L. amazonensis*, in the ear dermis. *L. amazonensis* infection in the C57BL/6 genetic background results in a moderate form of cutaneous leishmaniasis at the site of parasite inoculation (Additional file 8: Figure S6A). In these mice the clinical phenotype evolved according three phases. First, the pathophysiological process is mainly associated with a cutaneous lesion displaying an inflammatory aspect, erythematous oedema, then culminating with tissue ulceration necrosis, leading often to tissue loss and finally a persisting wound, which retains a very discrete inflammatory aspect (Additional file 8: Figure S6). The longitudinal analysis of the ear widths indicates that the lack of OPN increased the inflammatory grade of the ear lesion (Fig. 5a). Consistently with the distinct clinical phenotype between wild type and *opn* deficient mice, clinical observation at Day 48 *p.i*., showed a higher inflammation at the ear lesion level in the *opn* mutant mice (Fig. 5e, b and Additional file 8: Figure S6B). In addition, while lesions seemed resolved after Day 84 *p.i*. in the wild type mice, inflammatory, ulcerative and necrotic foci were still present in the ear lesions of the mutant mice (Fig. 5e, d and e respectively and Additional file 8: Figure S6). In contrast parasitic load increased with similar kinetics over time in the lesions of both *opn* wild type and mutant mice (Fig. 5b).

Parasitic load and clinical phenotypes were further evaluated by establishing three phenotypic windows corresponding to 0–20 days *p.i*., 20–50 days *p.i*. and over 50 days *p.i*. (Fig. 5c and d). While parasitic load remained similar over time between the wild type and mutant mice (Fig. 5d), significant differences in ear width were observed at the last phenotypic window (over 50 days *p.i*.) whereas lesions remained more severe in the *opn* mutant mice (Fig. 5c). It is possible that similar differences as the ones observed in vitro between *opn*^+/+^ and *opn*^−/−^ BMFs may also be present in vivo, earlier than at 30 days *p.i*.. The low limit rates of bioluminescence detection, prior to 30 days *p.i*., render difficult earlier in vivo parasite measurements.

### OPN moderates the transcription of the inflammatory response to parasites on sites of inoculation

**Fig. 6:**
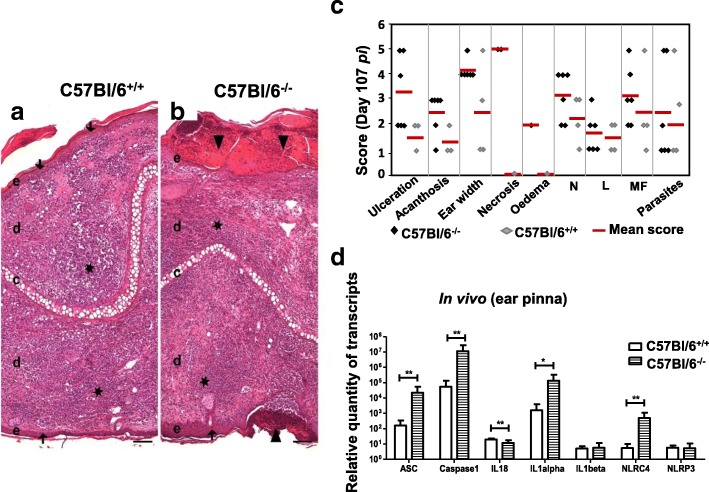
Histopathology and evaluation of the inflammasome transcripts on the site of parasite inoculation. **a**. Histological features of ear lesions in wild type C57BL/6^+/+^ and **b**. *opn* mutant C57BL/6^−/−^ mice at 107 days *p.i*.. Microscopic lesions (ulceration, acanthosis and inflammation) were more severe in C57BL/6^−/−^ than in C57BL/6^+/+^. Triangles ▼ : ulceration, stars ★ : inflamation, arrow ↓ : acanthosis, **c**: cartilage, **e**: epidermis, **d**: dermis, Hematoxylin and Eosin (H&E), original magnification × 10, scale bar: 100 μm. **C.** Histological scores from C57BL/6 wild type (black lozenges) and C57BL/6^−/−^ (grey lozenges) mice, post inoculated with 10^4^ *L. amazonensis* metacyclic promastigotes. Red bars indicate median values for each group. L = Lymphocytes, MF = Macrophages, N = Neutrophils (inflammatory). Number of mice studied: 6 KO; 4 WT. **d**. Ten thousand metacyclic promastigotes were inoculated in the ear dermis of C57BL/6 wild type (white columns) and *opn* mutant mice (columns with pattern). Data are from 3 representative mice for each strain at 107 days *p.i*. Relative quantity of transcripts is calculated by using the non-infected control tissues as the calibrator. Mean values and SDs are shown for each group. A Mann-Whitney test was performed to compare the fold changes between the two groups: *: *P* < 0.05; **P < 0.01

Microscopic evaluation of the sites of parasite inoculation at the last phenotypic window (day 107 *p.i*.) confirmed the severity of the local inflammatory phenotype in the mutant mice (Fig. 6). Indeed tissues on sites of inoculation of the mutant mice (C57BL/6^−/−^) showed ulceration, acanthosis, necrosis and inflammation (neutrophil and lymphocyte infiltration) to be more severe (Fig. 6b) than in the wild type mice (Fig. 6a and Additional file 9 FigureS7). Histological scores also were consistently higher in the mutant lesions (Fig. 6c). These data are in agreement with the higher scores of ear width and the persistent parasite burden observed in these mice (Fig. 5a, c). Recruitment of macrophages and neutrophils on the site of inoculation reflects the activation of the immune response. The consequence of this activation may well be the restriction of *L. amazonensis* parasites as previously demonstrated for *L. infantum* through a TLR2-dependent mechanism [40].

We then evaluated by RT-qPCR the expression of genes coding for cytokines and proteins implicated in the inflammation, in tissues from sites of parasite inoculation (ear pinna), in the C57BL/6 wild type and *opn*^−/−^ mice (Fig. 6d).

Low levels of transcripts coding for IL-1β and NLRP3 were observed in both wild type and *opn* mutant mice, indicating that seemingly *L. amazonensis* parasites contain the local inflammation response in vivo (Fig. 6d). In particular in the *opn* wild type mice *Asc, casp1*, *IL1-α,* and NLRC4, showed lower transcript accumulation in comparison with the *opn* mutant mice (Fig. 6d). The presence of different cell types on the dermal sites of parasite inoculation may account for the variability in the expression levels of the inflammasome genes observed in comparison with the infected macrophages. In the lesions of the dermis the inflammatory response triggered by the parasites implicates not only monocytes but also the cells of the dermis and the connective tissue (i.e. keratinocytes and fibroblasts) [41, 42].

The presence of the OPN moderates the expression of the inflammasome-related genes and this phenomenon is weakened by the absence of OPN in the mutant mice, similarly to our BMFs observations (Fig. 6d). It seems therefore that under the influence of OPN, while macrophages accumulated on the sites of injury in *Leishmania* infected sites, the parasites act by moderating the inflammasome transcripts. While the inhibition of the inflammasome transcripts by *L. amazonensis* parasites and the role playing by the osteopontin are intriguing; additional investigation is required i) to demonstrate if the OPN implication represents a direct effect or an effect requiring the regulation of other molecules and ii) to identify the in vivo cell types implicated and the exact mechanisms involved: thus novel strategies to control *L. amazonensis* parasites infection may be revealed.

### *L. amazonensis* parasites stimulate in vivo *opn* gene expression in the C57BL/6 mice

**Fig. 7:**
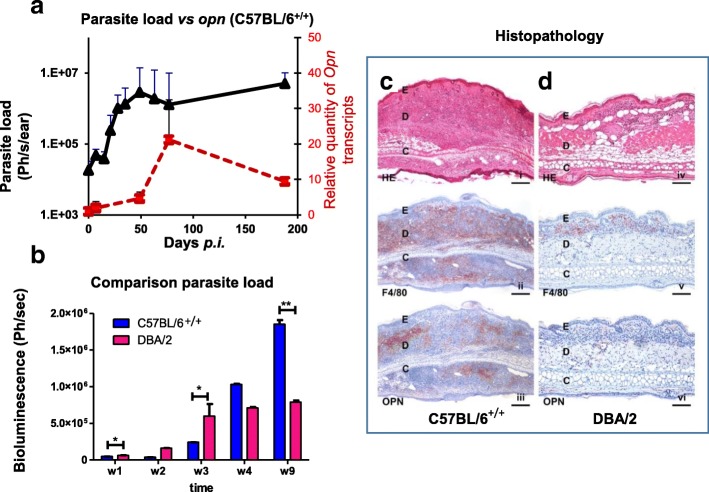
Comparative analysis of parasitic load and *opn* transcripts between C57BL/6 and DBA/2 mice in vivo. **a**. Parasitic load and *opn* gene expression in C57BL/6^+/+^ mice, in vivo. Matched evaluation of parasite load fluctuations (black line) by bioluminescence signal, expressed in photons/ sec /ear and RT-qPCR of the relative quantity of *opn* transcripts (red line) in sites of inoculation. Correlations between parasite load and *opn* transcripts were as follows: C57BL/6, R Correlation *P* = 0.0297 and DBA/2 mice, R Correlation *P* = 0.4618 (data not shown). At each time point a group of 3 representative mice on the basis of parasite load were tested. **b**. Matched evaluation of the fluctuations in parasite load (bioluminescence) in infection sites, during the first weeks *p.i.* with *L. amazonensis* of C57BL/6 (blue bars) and DBA (red bars) mice. Mean values are for 3 representative mice at each time point. A Mann-Whitney test was performed to compare the fold changes between the two groups: *P < 0.05; **P < 0.01. Non-parametric correlation tests (Spearman) were performed to compare Bioluminescence. **c-d**. Histopathological analysis of primary lesions of C57BL/6 (**c**) and DBA/2 (**d**) mice subcutaneously injected with 10^4^ *L. amazonensis* promastigotes in the right ear pinnae at 80 days *p.i*. (i) In C57BL/6, *L. amazonensis* elicited a severe inflammation within the ear dermis, H&E staining, original magnification × 4, scale bar: 250 μm. (ii) Many macrophages were present within the inflammatory infiltrate, F4/80 immunostaining, original magnification × 4, scale bar: 250 μm. (iii) OPN expressing cells were present within the lesion, OPN immunostaining, original magnification × 4, scale bar: 250 μm*.* (iv) In DBA2, a mild inflammation was noted at the site of inoculation, H&E staining, original magnification × 10, scale bar: 100 μm. (v) A low number of macrophages were present within the lesion, F4/80 immunostaining, original magnification × 10, scale bar: 100 μm. (vi) Very few OPN expressing cells were observed, OPN immunostaining, original magnification × 4, scale bar: 250 μm

In order to address the direct effect of *L. amazonensis* parasites on the *opn* gene expression, C57BL/6 mice infected with metacyclic promastigotes in the ear dermis were compared to DBA/2 similarly treated female mice, known to control parasite load and inflammation after *L. amazonensis* infection [11, 43]. Longitudinal evaluation of parasitic load was performed simultaneously for both strains, by measuring bioluminescence emitted from the LV79-luciferase parasites (Fig. 7a and b). While parasite load consistently increased up to 50 days *p.i*., *opn* gene expression followed with slower kinetics, with a pic at about 80 days *p.i*. in the C57BL/6 mice (Fig. 7a, red line). In contrast, in the DBA/2 strain, despite an increase of parasitic load (Fig. 7b, red bars) *opn* gene expression remained constantly low (data not shown).

At Day 190 *p.i. opn* transcripts were two fold decreased (compared to the pic values) while parasitic load remained high in the C57BL/6 mice (Fig. 7a). This is probably due to the end stage host response to the parasites, characterized by resorption of the inflammation and subsequent destruction of the cutaneous tissue. At this stage, immune cells, such as macrophages, expressing OPN are rare. In contrast, in DBA/2 mice *opn* transcripts remained low on the sites of parasite inoculation correlating with the absence of the host inflammatory response to *L. amazonensis* in this strain (data not shown).

At 80 days *p.i*. corresponding to the observed pic of the longitudinal analysis (Additional file 10: Figure S8A), *opn* transcripts were increased 20 fold in the ear pinna of the C57BL/6 mice (relative to the non-infected control values).

In the draining lymph nodes (LN) of the inoculation sites, *L. amazonensis* stimulated *opn* gene expression 10 and 6 fold in the C57BL/6 and DBA/2 mice respectively, relative to the control (Additional file 10: Figure S8B), indicating that the parasites trigger an immune response in both genetic backgrounds despite the phenotypic differences.

Histological evaluation of ear sections of C57BL/6 and DBA/2 mice, at Day 80 *p.i.* confirmed by H&E staining, the larger lesion widths observed in the C57BL/6 mice (Fig. 7c, i) in comparison with the inoculation sites of the DBA/2 strain (Fig. 7d, iv). Immunostaining with the anti-F40/80 antibody specific to a 160 kD glycoprotein, a MF marker, showed a balanced population of these cells colonizing the inoculation sites and accumulating at high numbers in the C57BL/6 mice (Fig. 7c, ii), while lower numbers were observed in the DBA/2 mice (Fig. 7d, v). Staining with anti-OPN antibody revealed a high protein content in the C57BL/6 mice, while residual cells expressed OPN protein in the DBA/2 lesions, (Fig. 7c, iii and d, vi, respectively). The low *opn* transcripts observed in the DBA/2 mice together with the presence of the relatively high parasitic load in the inoculation sites are in agreement with our data in the C57BL/6 mice whereas similar parasitic loads where observed in vivo in the presence as well as in the absence of OPN (Fig. 5b and d). However in the absence of *opn* while increased inflammatory grades of the ear lesions were observed in the C57BL/6 genetic background (Fig. 5a), the DBA/2 mice do not develop an inflammatory phenotype (Fig. 7d). This suggests that the OPN response to parasites is related to the strain-dependent immune response to *L. amazonensis*. Two hypothesis may be advanced for these data: a) OPN is implicated in the inflammatory response to *L. amazonensis* rather than in affecting parasite proliferation, in vivo. Thus the low numbers of focal accumulation of macrophages in the infected ear lesions of the DBA/2 in comparison with the C57BL/6 mice (F4/80 immunostaining Fig. 7d, v vs c, ii, respectively) is correlated with the low levels of *opn* gene expression observed in the DBA/2 mice (Fig. 7d, vi vs c, iii). b) Alternatively in the DBA/2 genetic background *opn* gene expression is specifically down regulated, reflecting the implication of this molecule in the control of the inflammatory response.

## Discussion

OPN plays many roles in the regulation of immune response on multiple levels. It contributes to the development of a variety of immune-mediated inflammatory diseases [44] and it regulates the host response to infection [45]. It was postulated that susceptibility of mice to *L. amazonensis* is contributed by multiple mechanisms. OPN is considered a Th1 cytokine [17]. Impaired Th1 responses were reported in *L. amazonensis* infected hosts [46], while a mixed Th1/Th2 response was shown to be maintained via an unknown mechanism in BALB/c susceptible mice inoculated in vivo with *L. amazonensis* [47].

We therefore evaluated the implication of this cytokine in the host response to *L. amazonensis* in vitro in BMFs and in vivo by inoculation of metacyclic promastigotes in the ear pinna of C57BL/6 wild type and *opn* mutant mice.

Our data provide evidence **i**) for the implication of OPN in the host response to parasites and report the up-regulation of *opn* gene expression by *L. amazonensis* in vitro in BMFs and in vivo in the C57BL/6 mice and **ii**) we showed that *L. amazonensis* parasites inhibit inflammasome-related transcripts in infected macrophages in vitro as well as on the inoculation sites in vivo*.* Indeed the parasites inhibit the expression of NLRP3 and NLRC4 inflammasomes in vitro in the BMFs and moderate the inflammasome response at least at the transcriptional level in vivo in C57BL/6 mice. Moreover despite an over two-fold stimulation of the *casp-1* transcripts, *IL-1β* expression was over 10 fold down-regulated in macrophages infected with *L. amazonensis* amastigotes*.* This phenomenon was moderated by the absence of *opn* in the mutant mice (Fig. 4) and highlighted by the presence of pyroptotic-like cells in infected *opn* mutant BMFs (Fig. 3 and Additional file 7: Figure S5). Additional experiments with double knock out models carrying inactivated inflammasome and *opn* genes, may provide new insights for the exact mechanisms.

*L. amazonensis* parasites stimulated *opn* gene expression and OPN protein (Fig. 2) but parasitic load (mean intensity) was contained, with an increase of three fold between 24 h and 48 h *p.i*. in the presence of OPN, (Fig. 1c), while in contrast, in the *opn* mutant mice, parasite numbers per cell increased over fivefold at 48 h *p.i*.. This suggests that this molecule is implicated in the host response to *L. amazonensis* by limiting parasite intensity, without however killing the parasites. OPN seems to participate in the host adaptation to the *L. amazonensis* parasites by inhibiting the inflammasome-related transcripts, as seen in the wild type mice. The lower parasite numbers found in these animals indicates that in the presence of OPN parasite growth is limited, which eventually contributes towards a defence mechanism against the parasites. However it is not clear at present if this is a direct or indirect effect of the OPN on parasite proliferation.

In a recent report was shown that *Leishmania* species including *L. amazonensis*, activated the inflammasome in BMFs and in vivo [48]. In these experiments, pre-treatment of macrophages with liposaccharide (LPS) or interferon-γ (IFN-γ) was performed prior to infections. LPS stimulates the immune response by inducing the generation of cytokines such as tumour necrosis factor (TNF)-α, interleukin-1 (IL-1) and IL-6, by a mechanism that is not well defined at present. Under these conditions, these authors, found that IL-1β contributed to induced nitric oxide synthase (iNOS)-mediated production of nitric oxide (NO). NO acts as a critical negative regulator of the NLRP3 inflammasome in human monocytes and mouse BMFs [49] and is known to play an important role in host resistance to infection, particularly to *Leishmania spp* parasites [50–52]. Enhanced susceptibility to *L. amazonensis* infection correlates with reduction of inflammatory responses accompanied by low levels not only of IFN-γ, LT-TNF (Lymphotoxin-tumour necrosis factor) but also of NO production [50]. Therefore the activation of the inflammasome reported after induction with IFN-γ and LPS of BMFs prior to *L. amazonensis* inoculation, may be mediated by the NO produced, which in turn kills the parasites.

In contrast to these findings, our data indicate that *L. amazonensis* inhibits the transcripts involved in NLRP3 and NLRC4 inflammasomes through inhibition of *IL-1β* and this effect requires the presence of OPN in the BMFs. As we identified inhibition of the inflammasome-related transcripts by *L. amazonensis*, we did not examine the levels of the corresponding proteins which are expected to be undetectable by Western blot analysis.

To note that in the female DBA/2 mice 17β-estradiol increases *L. mexicana* killing by enhancing the production of Nitric Oxide (NO), while pro-inflammatory cytokines do not seem to be involved [53]. The same authors found that parasite control in female DBA/2 mice was associated with higher production of IFN-γ. Interestingly OPN was reported to be a negative feedback regulator of NO synthesis in murine macrophages by mediating inhibition of iNOS expression and NO production [54].

The role playing by OPN in the host response to *L. amazonensis* revealed to be quit intriguing. It is known that OPN produced by macrophages inhibits NO production by suppressing iNOS expression [55, 56]. In activated MF by treatment with LPS and IFN-γ, induction of both iNOS and OPN transcripts was observed, suggesting that NO directly up-regulates the endogenous OPN in these cells after LPS and IFN-γ stimulation [56]. The mechanisms used by *Leishmania* parasites to evade killing by NO remain still largely unclear [57]. Although in this report we did not examine the expression of iNOS and NO, induction of the *opn* gene expression by the parasites together with the inhibition of the inflammasome transcripts in the presence of *opn* indicate a balance between the *trans* regulatory feed-back loop mechanism of NO and OPN. In contrast, in the absence of *opn* gene the increase of parasitic load and the pyroptosis-like phenotype observed may be the outcome of a dysregulation of the iNOS and NO production in response to parasites, requiring the OPN protein as part of the host-parasite interaction.

Finally, the impact of *Leishmania* on the inflammasome formation, IL-1β production and NO stimulation, although clearly was shown to be up-regulated after LPS and IFN-γ treatment of the cells, by Lima-Junior and coll., in our study in the absence of pre-treatment, this phenomenon was not observed. However it is to be noted that in the absence of pre-treatment these authors also detected reduced amounts of IL-1β production and caspase-1 cleavage in infected macrophages with the parasites and concluded that “in some instances *L. amazonensis* modulates and perhaps inhibits inflammasome activation” [48]. Indeed our data demonstrate such inhibition of the inflammasome transcripts by the parasites that can be explained by the absence of stimulation of the cells. Similarly in a recent report a protective role for OPN after viral infection was identified to be correlated with the control of the inflammasome and the restriction of apoptosis in the murine CNS [58].

The importance of the implication of the inflammasome affecting innate and adaptive immune responses is highlighted by several recent reports in the context of infection, autoimmune disorders and vaccination [59–66]. However the existing methods to identify inflammasome inference in various inflammatory conditions are limited in the detection of the transcripts or proteins known to be part of this multimeric protein complex. Recently it was reported an in vivo imaging of the inflammasome activation in MFs [67]. Additional studies set to combine molecular methods with in vivo real time imaging will greatly contribute in a better comprehension of the fate of *Leishmania* parasites-infected cells.

During infection, pyroptosis can be beneficial to the host but it can be harmful during overwhelming infection as seen by the burst of the macrophages in vitro. The molecular mechanisms of the virulence strategy employed by *L. amazonensis* parasites remain not clear. Inhibition of the inflammasome may protect parasite replication in the host cells but may be detrimental for perpetuating infection.

## Conclusions

Our data are in agreement with the implication of the osteopontin in containing parasite proliferation in the host cells, while in contrast in its absence parasite crowding increases with as consequence a pyroptotic-like cell death of the macrophages. Our results reveal that *L. amazonensis* parasites contain the inflammasome transcripts in vitro in the BMFs and in vivo, in the C57BL/6 mice resulting in a restriction of parasite intensity. OPN is implicated in the host-response to *L. amazonensis* by moderating in particular the inhibition of the *IL-1β* transcripts. These data suggest that this molecule plays a role in the host-response to *L. amazonensis* and has the potential to be further evaluated as a target for *Leishmaniasis* control and eventually may be useful to improve vaccination strategies against the parasites.

## Methods

### Mice and ethical statement

Pathogen-free 6–8 weeks wild type C57BL/6 and DBA/2J female mice were purchased from Charles River (Europe, CRL France). *Opn* mutant congenic mice (B6(Cg)-Spp1^tm1Blh^/J) backcrossed 10 times to a C57BL/6 genetic background [68], were obtained from the Jackson Laboratories (Bar Harbor, ME) and are abbreviated C57BL/6^−/−^ in the manuscript. All mice were bred under specific pathogen–free conditions, in the animal facility of Institut Pasteur, and littermates destined to be inoculated with *L. amazonensis* parasites were randomly assigned to experimental treatment groups and housed in the A3 animal facility. All experimental protocols were ethically approved by the Institutional Committees on Animal Welfare at Institut Pasteur (CETEA n°: 2013–0014) and conducted under strict accordance with the European guidelines (Directive 2010/63/EU) for animal care and use. EM is authorized to perform experiments on vertebrates from the Paris Department of Veterinary Services, (DDSV) and she was responsible for all experiments conducted personally or under her supervision under strict consideration of the animal welfare ethics rules.

### Anesthesia and euthanasia

Anesthesia procedures are as described in the next paragraph section.

Animals used for the experimental procedures in this work were euthanized by carbon dioxide (CO_2_) delivery from a compressed CO_2_ gas cylinder directly in the animals’ home cage to reduce stress, as recommended. Euthanasia was accomplished by slow exposure to increasing levels of CO_2_, displacing approximately 10–30% of the cage volume per minute, outside the animals’ housing room. Flow was kept for 2 min and the animals were maintained in the same cage for five additional minutes. Animals were bred taking in consideration the animal numbers necessary for our studies. However when this was necessary remaining animals were euthanized using the same procedure as described. For RNA preparations animals were sacrificed by cervical dislocation after light anesthesia by intraperitoneal injection of Pentobarbital (50 mg/kg).

### Preparation and inoculation of *L. amazonensis* parasites

*Leishmania amazonensis* strain LV79 (WHO reference: MPRO/BR/72/M1841) was used for parasite preparation under experimental procedures previously described [69–71]. In brief, 10^6^ wild type LV79 amastigotes were injected subcutaneously in the hind footpad of Swiss nude mice. Lesions containing the parasites were excised 2 months after and purified, as described [69, 72]. LV79 at the amastigote developmental stage were used to inoculate BMFs cultures at a MOI (Multiplicity of infection) of 4:1. LV79 metacyclic promastigotes were prepared from amastigotes carrying the firefly luciferase gene into the 18S rRNA locus of the nuclear DNA of *Leishmania amazonensis* LV79 strain [69]*.* After the initial amastigote differentiation into promastigote, parasites were grown at 26 °C in supplemented M199 medium, as previously described [73]. Mammalian infective-stage metacyclic promastigotes were isolated from 6 days stationary phase cultures using a discontinuous density gradient [74]. Increased yield (≥95%) of metacyclic promastigotes was obtained as demonstrated by percentage counts after fixation and Giemsa coloration of the parasites [75, 76], as well as by RTqPCR for the presence of *sherp* transcripts (E. Giraud under review). Ten thousand metacyclic promastigotes into 10 μl of PBS (Dulbecco’s phosphate buffer saline) were injected in the ear dermis of C57BL/6^+/+^, C57BL/6^−/−^ and DBA/2 mice as previously described [11, 77]. The mice were anesthetized by administration of ketamine (120 mg/kg Imalgène 1000, Merial, France) and xylazine (4 mg/kg; Rompun 2%, Bayer, Leverkusen, Germany) by intraperitoneal injection. Each mouse was tagged at the collateral ear for identification. Clinical phenotypes were evaluated as the range of the lesion size on the site of inoculation and compared to the non-inoculated contra-lateral ear as previously described [69], by a direct reading using Vernier calliper (Thomas Scientific, Swedesboro, NJ) and expressed as ear thickness.

### Bone marrow-derived macrophages generation and infections

Macrophages were differentiated from bone marrow cells isolated from the tibia and femurs of 6–8-weeks-old C57BL/6^+/+^, C57BL/6^−/−^ and DBA/2 mice as previously described [29, 72]. In brief, bone marrow cells were suspended in PBS-Dulbecco, Ca^++^, and Mg^++^ on ice. After centrifugation (300 g, 10 min, 4 °C) and red blood cell elimination with NH_4_CL 0.87%, pellets were re-suspended in complete DMEM medium (Gibco, Life Technologies) containing 10% FCS, 50u/μg Penicillin/Streptomycin, 50 mM 2-mercaptoethanol). Cells were counted and put in culture in the presence of rm-CSF-1 (ImmunoTools) at a density of 7.5 × 10^6^ cells/100 mm Falcon dish and placed at 37 °C (94% air, 7.5% CO_2_). On day 7, macrophages were inoculated with the *Leishmania amazonensis* amastigotes (strain LV79), freshly isolated from footpad lesions of Swiss nude mice as previously described [78], at a MOI 4:1 (parasites: MF). Cell cultures were then incubated at 34 °C for 24 or 48 h and cells were lysed for total RNAs preparation or placed on glass micro slides for fluorescent immunostaining with antibodies against LAMP-1, osteopontin and *Leishmania*. For evaluation of intracellular and extracellular OPN protein concentrations, by Western blot and Elisa assay differentiated BMF cells were infected with axenic amastigotes as described [29]. For these experiments the LV78 strain (MPRP/BR/72/M1845) was used, belonging to the same complex of *L. amazonensis* than the LV79 strain. For the generation of axenic amastigotes, parasites were subjected to host-free differentiation conditions by simultaneous pH (7.4 to 5.5) and temperature (26 °C to 34 °C) shift of the fully supplemented M199 in the presence of 20% FBS and titrated to pH 5.5 with succinic acid as previously described [37] and allowed to differentiate for 7 days. At 24 h, and 48 h post infection, the culture medium was removed and kept for the ELISA assay. For the Western blot analysis cells were washed three times with 4 ml PBS and lysed in 200 μl RIPA buffer (150 mM sodium chloride, 1.0% Triton X-100, 0.5% sodium deoxycholate, 0.1% SDS (sodium dodecyl sulfate) 50 mM Tris, pH 8.0. To note that the permissive temperature for the surviving and multiplication of the *L. amazonensis* amastigotes is 34 °C [79], similar to the dermis temperature on the sites of parasite infection by the sandfly (lower than the body’s 37 °C).

### SDS PAGE and immunoblotting

SDS-polyacrylamide gel electrophoresis (SDS-PAGE) was performed by the method of Laemmli (1970) [80]. For immunoblotting, 15 μg of protein extracts were loaded on 12% SDS-PAGE gel and once electrophoresis completed, proteins were transferred on a nitrocellulose filter (Hybond C, Amersham Biosciences) and immunoblotting was performed with the use of ECL Plus (enhanced chemiluminescence) (GE Healthcare) according to the manufacturer’s instructions. OPN and actin were detected sequentially after stripping the blot for 10 min RT with stripping buffer (200 mM glycine, 1% SDS, pH 2.5). Goat anti-mouse OPN antibody (AF808, R&D Biosystems) was used at a dilution of 1:750 in TBS buffer. Donkey anti-goat IgG (H&L, ab205723, Abcam) Horse Radish Peroxidase (HRP) conjugated was used as a secondary Ab at a dilution 1:10000. Anti-actin Antibody (MAB 1501, clone C4, Merck, Millipore) at a 1:2000 dilution was used for detection of the actin protein and a goat anti-Mouse IgG (H + L) secondary Antibody (0.8 mg/ml), HRP conjugate (Invitrogen) at 1:10000 dilution.

### Quantification of secreted OPN by enzyme linked immunosorbent assay (ELISA)

Secreted OPN was evaluated in the media from BMF cells infected or non-infected with *Leishmania amazonensis* parasites by a quantitative sandwich enzyme immunoassay method using the Quantikine mouse osteopontin ELISA kit (R&D Systems, Cat N° MOST00) as described by the manufacturer. Briefly, a polyclonal mouse OPN specific antibody is pre-coated onto a microplate. Samples, standards and controls are added into the wells and mouse OPN present is bound on the immobilized antibody. Following 2 h incubation at room temperature, unbound substances are washed and an enzyme-linked mouse OPN specific polyclonal antibody is added to the wells and incubated for 2 h at room temperature. After removing any unbound antibody-enzyme reagent by five series of washes, a substrate solution is added to the wells and incubated for 30 min at room temperature in the dark. The enzyme reaction yields a blue color that turns yellow after adding the STOP solution. The color intensity corresponding to the amount of mouse OPN bound in the initial step was read at OD 450 with a correction wavelength set at 570 nm in an absorbance microplate Reader ELx800™. Standard curve was prepared by reconstitution of mouse OPN Standard (2500 pg/ml) following by serial dilutions at 8 concentrations in total, from 2500 pg/ml to 39 pg/ml, and 0 pg/ml. Samples were serially diluted to 1:3, 1:9, 1:27, to 1:81 in the buffer provided in the kit. All buffers used are as described and supplied in the kit (https://www.rndsystems.com/products/mouse-rat-osteopontin-opn-quantikine-elisa-kit_most00).

The ELISA scores of the samples were submitted to a quantitative analysis using a Four Parameter Logistic (4PL) curve-fit suitable for calculating concentrations from symmetrical sigmoidal calibrators as described (https://www.myassays.com/four-parameter-logistic-curve.assay). The standard data points (concentration vs. measurement) are plotted on log-log axes and a 4PL is made through the points. The concentrations of the samples are determined taking in consideration the specific dilution factors applied.

### Flow cytometry of macrophage-restricted markers

The differentiation of macrophages from the bone marrow progenitors isolated from the DBA/2, C57BL/6^+/+^ and *opn* mutant, C57BL/6^−/−^ mice were examined by Flow cytometry for the coordinated expression of monocyte/macrophage lineage specific gene expression of surface molecules including CD11b, CD115, CD11c (cluster of differentiation), MHC-II and the F4/80 antigen expressed by the Lymphocyte antigen (Ly-71), using the corresponding antibodies [81, 82]. Flow cytometry was performed on a Gallios flow cytometer (Beckman Coulter) and the data were analysed using the Kaluza software package (Beckman Coulter) as previously described [72]. All three strains of mice showed similar cell surface molecules designating the macrophage lineage as follows: macrophage lineage-specific: CD11b^High^ F4/80^High^, CD115^High^, while surface-specific markers for the dendritic cell (DC) lineage, were negative or low (CD11c^−^, MHC-II^Low^) (Additional file 1: Figure S1).

### In vivo luciferase-expressing *L. amazonensis* bioluminescence imaging

Animals were followed for clinical phenotypes (usually weekly) and parasite proliferation at different time points after infection, ranging from Day 16 to Day 100. Parasite loads were evaluated by bioluminescence imaging as previously described [69]. In brief, mice were given a solution of the luciferase substrate luciferin (*i.p*. 150 mg/kg, D-Luciferin potassium salt, SYNCHEM OHG, Germany). 25 min following luciferin injection mice were anaesthetized for 5 min in a 2.5% isoflurane atmosphere (Aerane, Baxter SA, Maurepas, France) and then placed into the imaging chamber (IVIS™ Imaging System 100 series, Xenogen). Emitted photons acquisition from the sites of inoculation (regions of interest, ROI) was acquired by a charge-coupled device camera delimiting the surface of the entire ear pinna. The same ROI was examined for all mice at all time points. Total photon emission was expressed in photons/sec/ROI. Median bioluminescence values and SD were calculated for each experimental group. At designated time points post inoculation, 3 representative mice from each group selected for their median bioluminescence and SD values, similar to the entire corresponding group were sacrificed and ear pinnae and draining lymph nodes were removed for further process of total RNA extraction or for histology [77]. Contralateral tissues non-injected and control mice groups were analysed in parallel.

### Immunofluorescence labelling of BMFs

Bone marrow derived macrophages were placed on glass slides (CML France) in 24 wells plates and inoculated or not with *L. amazonensis* amastigotes (MOI 4:1). Cell cultures were placed at 34 °C (94% air, 7.5% CO_2_) for 24 h and 48 h. Culture media were then removed and cells were washed with 1 ml 1xPBS (Dulbecco) without Mg^++^ and Ca^++^ at 34 °C. Cells were fixed on glass slides with 1 ml /well 4% parafolmahedyde (PFA) in PBS pH 7.4 for 1 h at room temperature, permeabilized with saponin (25 mg/ml). Incubation with the primary antibodies was as follows: 10 μg/ml of the amastigote specific mAb 2A3–26-biot, and anti-OPN (goat IgG) 7 μg/ml (R&D Systems, France) or LAMP-1/CD107a monoclonal antibody specific for the lysosomal-associated membrane protein 1 (LAMP-1) of the parasitophorous vacuole (rat IgG2a FITC, Invitrogen, CA). Revelation was performed with 1.5 μg/ml streptavidin conjugated to Texas Red (Molecular Probes, Cergy Pontoise, France) for the *Leishmania* parasites, with donkey anti-goat FITC (fluorescein isothiocyanate fluorochrome, sc2024, Santa Cruz Biotechnology) for osteopontin and donkey anti-rat-FITC (LS-C351178, CliniSciences, FR) for LAMP-1. The incubation at room temperature was for 30 min with the first antibodies and after three washes with PBS/saponin, 30 min with the secondary antibodies. Glass slides were then mounted with Hoechst 33342-containing Mowiol 4.88 (Calbiochem). Hoechst labelling of the DNA allowed visualization of both host cell and amastigote nuclei. Epifluorescence microscopy signals were detected with a Zeiss Axioplan 2 upright microscope monitored by the Zeiss AxioVision Rel. 4.8.2 image acquisition software (Carl Zeiss International). Mean protein densities were annotated using the digital high quality images acquired by the AxioVision software tool. An average of 10 different cells were analysed in at least three different fields. Values are expressed in density units/msec of 20μm^2^ *opn* stained areas/cell. Statistical analyses are performed with the Mann Whitney test.

For the phenotypic measurements of the BMFs we have used multidimentional acquisition of at least 4 different microscopy fields, using the MosaiX application (40x magnification) which permits to scan large areas of the slides creating a digital image. This enhanced quality image serves to navigate the samples and provides a basis for the analysis.

### Histopathology

Ear pinna and the draining lymph nodes were sampled, fixed in 4% formalin, and then embedded in paraffin and 5 μm sections were stained with haematoxylin and eosin. Each microscopic change: ulceration (i.e. breach of the continuity of skin, with sloughing out of inflamed necrotic tissue), acanthosis (i.e. hyperplasia and thickening of the epidermis), necrosis (i.e. mass of eosinophilic tissue and cell debris replacing the epidermis or dermis), oedema *(*i.e. collection of pale fluid within the interstitium) and presence of parasites were scored semi-quantitatively using a five-scale scoring system (1: minimal, 2: mild; 3: moderate, 4: marked, 5; severe). Median values were compared between inoculated sections and control contralateral tissues. Immunostaining with anti-OPN (AF808, R&D systems, Minneapolis, MN, USA) and anti-F4/80 macrophage-specific antibodies (MAB 5580, R&D systems) was also performed to estimate neutrophils and macrophages infiltration.

### RNA and DNA extraction

RNA preparation was performed in tissues isolated from three representative mice at various time points [77]. Ears and draining lymph nodes were fragmented with the Precellys 24 System and total RNAs were prepared with the RNeasy Plus Mini Kit (Qiagen, SAS, France) following the manufacturer’s instructions. 5 × 10^6^ BMFs were lysed by a tissue grinder with sterile pellet pestle mixer (Kontes, Thomas Scientific) and total RNAs were extracted with RNeasy Plus Mini Kit (Qiagen) as instructed by the manufacturer. RNA quantification and quality evaluation was done by measurement of Optical Density in a Nanodrop ND-1000 micro-spectrophotometer (ThermoFisher Scientific) [83]. DNAs were isolated from tissues or from amastigotes using the DNeasy Tissue Kit (Qiagen) under the manufacturer’s protocol.

### Real-time quantitative PCR

Reverse transcription of 1 μg of total RNAs to first strand cDNA was done using random hexamers (Roche Diagnostics) and the MMLV-RT reverse transcriptase (Moloney Murine Leukemia Virus, Invitrogen Life Technologies). Relative quantification of the genes of interest was performed in a final volume of 10 μl per reaction in white ultraAmp 384 well PCR plates (Sorenson, Bioscience, Salt Lake City, UT, USA) on a LightCycler® 480 system (Roche Diagnostics, Meylan, France). Briefly, 1 μl of cDNA or DNA was added to 9 μl of a master mix containing 5 μl of SYBR Green (QuantiTect SYBR Green Kit, Qiagen) and 4 μl of nucleases-free water with primers at a final concentration of 0.5 μM (Guaranteed Oligos™, Sigma-Aldrich). The PCR program included 40 cycles of denaturation at 95 °C for 10 s, annealing at 54 °C for 25 s and extension at 72 °C for 30 s. SYBR Green fluorescent emission was measured at the end of the elongation step. Crossing Point values (Cp) were determined by the second derivative maximum method of the LightCycler® 480 Basic Software. Raw C*p* values were used as input for qBase program for Biogazelle qBase program for qPCR data management and analysis [84]. For normalization eleven candidate control genes including *g6pd*, *gapdh*, *h6pd*, *hprt*, *ldha*, *nono*, *pgk1*, *rpl19, rpIIe, tbp* and *ywhaz* were tested with the geNorm [85] and Normfinder programs [86]. *Hprt* (hypoxanthine–guanine phosphoribosyl transferase) and *ywhaz* were the most consistently expressed in the BMFs and were selected as reference genes for the normalization of the gene expression levels. *Ldha* and *nono* were selected as the most stable reference genes for the C57BL/6 and DBA/2 lymph nodes. The primer sequences used were as follows: i) reference genes: *hprt* F- (5’-ATTAGCGATGATGAACCAG-3′), R- (5’-CTTGAGCACACAGAGGG-3′); *ywhaz* F- (5’-GTTACTTGGCCGAGGT-3′), R- (5’-GGAGTTCAGGATCTCGT-3′); *ldha* F- (5’-TCCTGGTGTGACTGGG-3′), R- (5’-CCAATAACGGCCACAAG-3′); *nono* F- (5’-AAAGCAGTTAGAACTCAGG-3′), R- (5’-GGCCCATCCGTATCTC-3′); *tbp* F- (5’-CCTATGACCCCTATCACT-3′), R- (5’-GTCCGTGGCTCTCTTAT-3′); ii) target genes: s*pp*1 (FL-*opn*) F-: (5’-TCTGATGAGACCGTCACTGC-3′), R- (5’-CCTCAGTCCATAAGCCAAGC-3′); i-*opn* F- (5’-GCCTGTTTGGCATTGCCTCCTC-3′), R- (5’-CACAGCATTCTGTGGCGCAAGG-3′); CD44 F (5’-TATCTCCCGGACTGAGG-3′), R- (5’-AGGCATTGAAGCAATATGT-3′); *casp*3 F- (5’-AGCTTTGTGTGTGTGAT-3′), R- (5’-CTGTCTGTCTCAATGCC-3′); p*ycard* F- (5’-TGCTTAGAGACATGGGC-3′), R- (5’-CCACTTCTGTGACCCT-3′); *nod2* F- (5’-AACAAACTCACGGATGC-3′), R- (5’-CCTTATCACCCACGCT-3′); *naip5* F- (5’-CATCCGGGTGAAGAGG-3′), R- (5’-GGGAACCACTTGGCAT-3′); *il-1β* F-(5’-AGGCAGGCAGTATCAC-3′), R- (5’-CACACCAGCAGGTTATC-3′) *casp1* F-(5’-ATCTGTATTCACGCCCT-3′), R- (5’-TGCTTCCTCTTTGCCC-3′); *nlrp3* F- (5’-GACTTTGGAATCAGATTGCT-3′), R- (5’-GGGTCCTTCATCTTTTCAC-3′); *nlrc4* F- (5’-AACTGGATTGCTTGGC-3′), R- (5’-AGTCAAAGCGTCCCAC-3′). The relative expressions of the *opn* gene and of the genes implicated in the inflammasome multicomplex were calculated as the *n*-fold difference in expression in the *Leishmania* infected BMFs in comparison with gene expression values from *Leishmania* unexposed cultures. Similarly, the evaluation of the inflammasome gene expression in the ear lesions was expressed as a ratio of transcript levels in the C57BL/6 ^−/−^ mice versus the wild type C57BL/6^+/+^ ear lesions, both inoculated in vivo with the parasites. *Leishmania* parasites were quantified with the gene target primers (*ssrRNA*) F- CCATGTCGGATTTGGT and R- CGAAACGGTAGCCTAGAG [11].

### Statistical analysis

Parasites numbers and parasite comparative quantification in the BMF cells are evaluated by “Mean parasite intensity” referring to the arithmetic mean of the number of individual parasites per infected host. Non-infected cells are not taken in consideration. “Prevalence” refers to the percentage of infected hosts and thus provides information of the relative sizes of the host cells in the study (infected and uninfected). In contrast, “crowding” is defined as the intensity from the parasite’s point of view. Mathematically, while “mean host intensity” is the sum of parasites per infected hosts, “mean crowding” is the sum of the crowding values (parasites living in a host) divided by the number of parasites [87]. In other words “Mean crowding” describes intensity values averaged across parasite individuals and represents the value of the number of parasites that they can live in a host cell [88]. Parasite quantification and comparisons of parasite loads, mean intensity and parasite crowding between macrophages from C5BL/6^+/+^ and C57BL/6 opn^−/−^ strains were performed using the Quantitative Parasitology (QP3.0) statistical software tool [53]. Comparison of mean intensities was done by a Bootstrap Test [89]. Density dependent character of parasites was described by Crowding, taking *p*-values at Cl (Confidence limit 95%) [89]. A Mann-Whitney test was used to compare transcript relative expression between macrophages from C5BL/6^+/+^ and C57BL/6^−/−^ BMFs. The p values were calculated with a Mann-Whitney test to compare ear bioluminescence, ear width, and the transcript relative expression obtained by RTqPCR between the control group and the group inoculated with parasites (*: *p* < 0.05; **: *p* < 0.001; ***: *p* < 0.0001).

## Additional files

Additional file 1: **Figure S1.** FACS analysis of BMF. **A**. Macrophage surface cell markers and **B**. Dendritic cells surface markers. Cells were analysed by FACS after differentiation of bone marrow monocytes to the macrophage cell lineage as described in the Methods section, at Day 6 corresponding to the Day of infection with the parasites. (PDF 312 kb)

Additional file 2: **Table S1.** Comparative statistical evaluation of parasite infectivity in BMF. Data are analysed by the Quantitative Parasitology software (QP3.0) [2] and are illustrated on Fig. 1 in the text. (PDF 13 kb)

Additional file 3: **Figure S2.** Schematic representation of the *opn* gene and mRNA structure. SP: Signal peptide; Fl: Full length OPN (in red PCR primers used) and iOPN: intracellular OPN (in green PCR primers used). (PDF 226 kb)

Additional file 4: **Figure S3.** Real-Time qPCR of CD44 in macrophages in response to *L. amazonensis.* No differences in the CD44 OPN receptor gene expression were observed at 24 h *p.i.* or 48 h *p.i*. in C57BL/6^+/+^ wild type and mutant mice (C57BL/6^−/−^). (PDF 164 kb)

Additional file 5: **Table S2.** In situ detection scores of infected (INF), Non-Infected (Non-INF) and Pyroptosis-like (INF-PYR) BMFs. Numbers of cells correspond to counts of at least 4 different image fields under grilles of immunofluorescence slides (40x magnification) using epifluorescence microscopy. Parasites, vacuoles and nuclei were labelled and evaluated using ApoTome and MosaiX software as described in the Methods section. Data are presented as graph on Fig. 3a. (PDF 166 kb)

Additional file 6: **Figure S4.** Host inflammasome-related and innate immune response transcripts in BMF infected with *L. amazonensis* parasites. Evaluation by qRT-PCR of transcript modulation isolated from BMF infected with *L. amazonensis* amastigotes of C57BL/6^+/+^ mice and *opn* mutant (C57BL/6^−/−^) mice (columns are as indicated in the legend): Inflammasome-related markers (*P* values WT vs KO) are CASP1 (*P* = 0.0317). Apoptosis markers CASP3 (*P* = 0.0278) and ASC (ns)**.** Parasite sensors NOD2 (ns) and NAIP5 (*P* = 0.05). Statistics are calculated from the mean of relative mRNA expressions at 24 h and 48 h *p.i*., versus the control values of the non-infected cells at each time point. (PDF 247 kb)

Additional file 7: **Figure S5.** BMF cell phenotypes in the presence and in the absence of osteopontin. The pyroptosis-like phenotype is observed in the absence of osteopontin indicating the implication of this protein in the cell adaptation to parasites. Immunostaining procedures are described in the Methods section. N: nuclei, 100x magnification. (PDF 215 kb)

Additional file 8: **Figure S6.** Evolution of representative ear phenotypic lesions at Day 48 and 104. **A**. C57BL/6 wild type phenotypic scores at Day 48 *p. i.*: 8/10 (80%) mild inflammation. At Day 104: 4/10 (40%) moderate to severe inflammation and 6/10 (60%) tissue disrupted at the level of infection. **B**. C57BL/6^−/−^ mice phenotypic scores at D48: 11/11 (100%) severe inflammatory lesions. At Day104: 7/11 (64%) destructive inflammatory lesions and 4/11 (36%) damaged tissue. (PDF 611 kb)

Additional file 9: **Figure S7.** Mean Histopathology scores. Mean values are from C57BL/6 wild type (green bars) and C57BL/6^−/−^ (red bars) mice, post inoculated with 10^4^ *L. amazonensis* metacyclic promastigotes. **A**. Tissue inflammation: N = Neutrophils; L = Lymphocytes, MFs = Macrophages. **B**. Tissue destruction. Number of mice studied: 6 KO; 4 WT. Unpaired t test with Welch’s correction, one tailed *P*-values. (PDF 270 kb)

Additional file 10: **Figure S8.** *Opn* gene expression in C57BL/6 and DBA/2 mice in vivo. Real-time qPCR of *opn* transcripts in C57BL/6 (blue bars) and DBA/2 mice (red bars) at day 80 *p.i*. corresponding to the pic of *opn* transcripts observed (**A**) in the ear pinna (see Fig. 7a) and (**B**) in draining lymph nodes. (PDF 16 kb)

## Abbreviations

ASC or PYCARD: Apoptosis-associated speck-like protein containing a CARD
BMF: Bone marrow-derived Macrophage
CASP1: Caspase 1
DC: Dendritic cell
IFN-γ: Interferon gamma
IL18: Interleukin 18
IL-1α: Interleukin 1 alpha
IL-1β: Interleukin 1-beta
iNOS: Nitric oxide synthase
L.: *Leishmania*
LN: Lymph nodes
LPS: Liposaccharide
LT-TNF: Lymphotoxin-tumor necrosis factor
NLRC4: NLR family CARD domain-containing protein 4
NLRP3: NOD-like receptor family, pyrin domain containing 3
NO: Nitric oxyde
OPN: Osteopontin
Th1: T helper 1
