# Supplementary figures and images for: Osteopontin in the host response to Leishmania amazonensis
Source: BMC Microbiol. 2019 Feb 8;19:32. doi: 10.1186/s12866-019-1404-z (PMC6368773; doi:10.1186/s12866-019-1404-z)

## FACS analysis of BMF

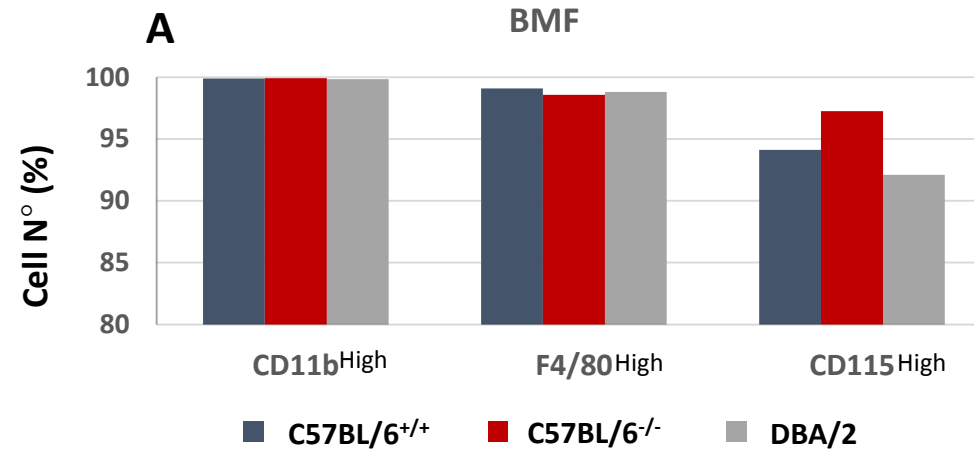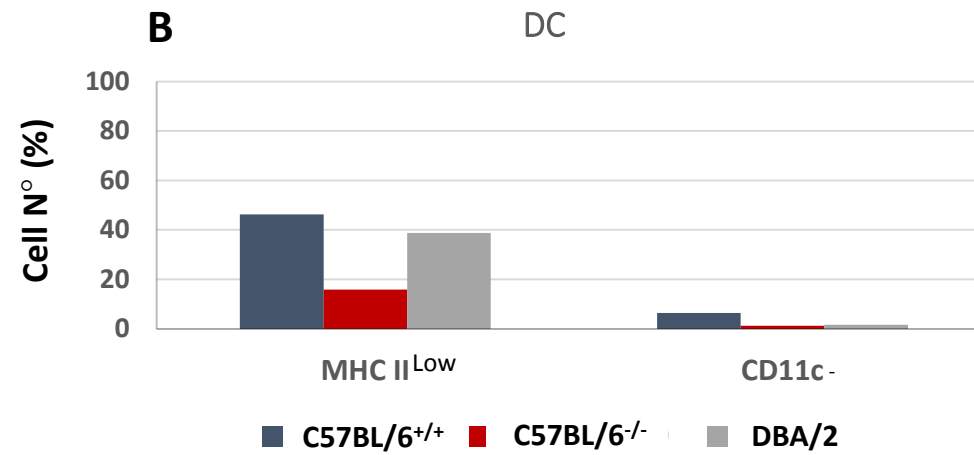

Supplement: Supplementary file 1 — Figure S1. FACS analysis of BMF. A. Macrophage surface cell markers and B. Dendritic cells surface markers. Cells were analysed by FACS after differentiation of bone marrow monocytes to the macrophage cell lineage as described in the Methods section, at Day 6 corresponding to the Day of infection with the parasites. (PDF 312 kb) [file 12866_2019_1404_MOESM1_ESM.pdf]

## *Opn* gene structure & mRNA

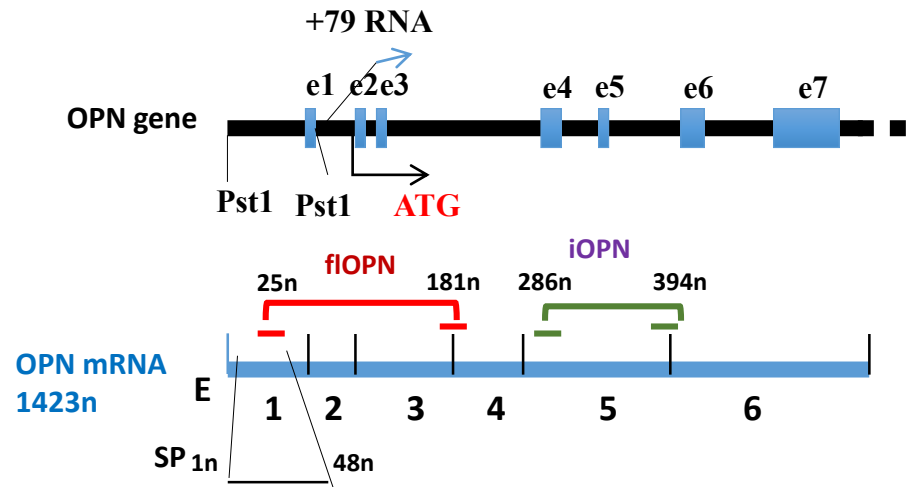

Fl: Full Length opn; iOPN: intracellular OPN

Supplement: Supplementary file 3 — Figure S2. Schematic representation of the opn gene and mRNA structure. SP: Signal peptide; Fl: Full length OPN (in red PCR primers used) and iOPN: intracellular OPN (in green PCR primers used). (PDF 226 kb) [file 12866_2019_1404_MOESM3_ESM.pdf]

### CD44 in response to *L. amazonensis*

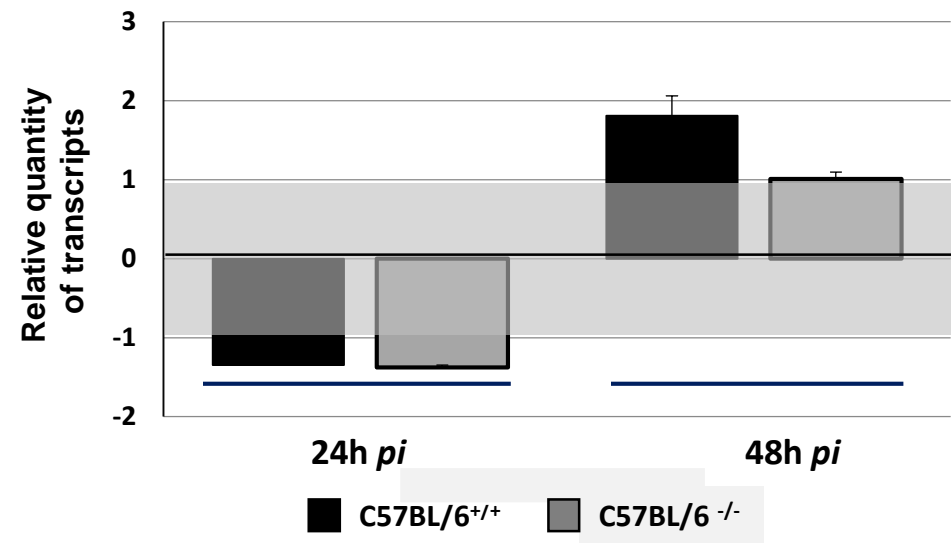

Supplement: Supplementary file 4 — Figure S3. Real-Time qPCR of CD44 in macrophages in response to L. amazonensis. No differences in the CD44 OPN receptor gene expression were observed at 24 h p.i. or 48 h p.i. in C57BL/6+/+ wild type and mutant mice (C57BL/6−/−). (PDF 164 kb) [file 12866_2019_1404_MOESM4_ESM.pdf]

## Expression of inflammasome-related transcripts

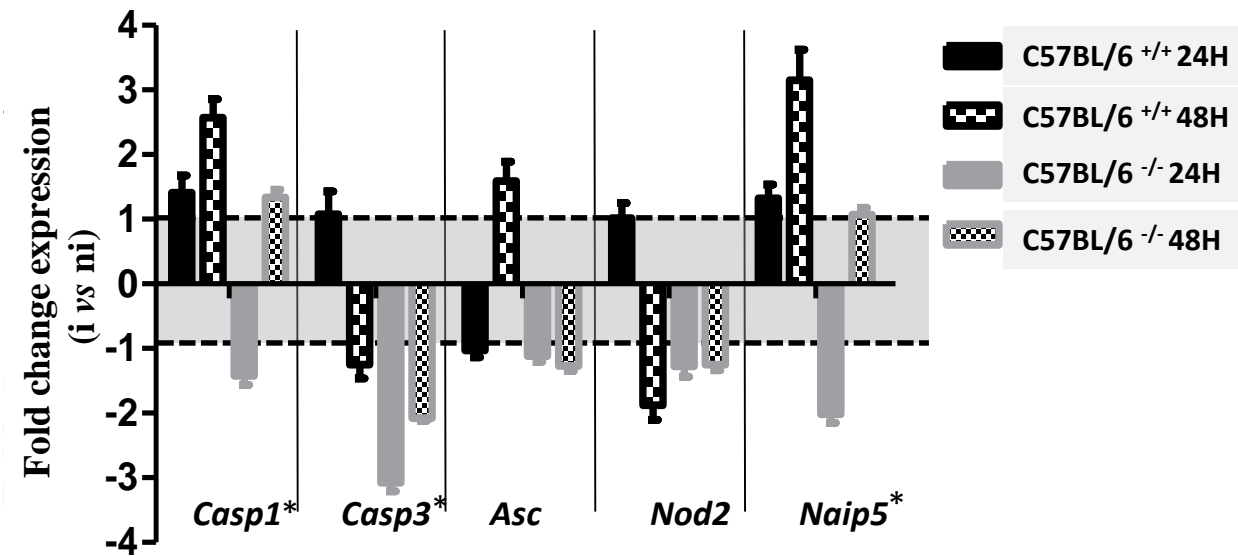

Supplement: Supplementary file 6 — Figure S4. Host inflammasome-related and innate immune response transcripts in BMF infected with L. amazonensis parasites. Evaluation by qRT-PCR of transcript modulation isolated from BMF infected with L. amazonensis amastigotes of C57BL/6+/+ mice and opn mutant (C57BL/6−/−) mice (columns are as indicated in the legend): Inflammasome-related markers (P values WT vs KO) are CASP1 (P = 0.0317). Apoptosis markers CASP3 (P = 0.0278) and ASC (ns). Parasite sensors NOD2 (ns) and NAIP5 (P = 0.05). Statistics are calculated from the mean of relative mRNA expressions at 24 h and 48 h p.i., versus the control values of the non-infected cells at each time point. (PDF 247 kb) [file 12866_2019_1404_MOESM6_ESM.pdf]

**BMF cell phenotypes  
in the presence and in the absence of osteopontin**

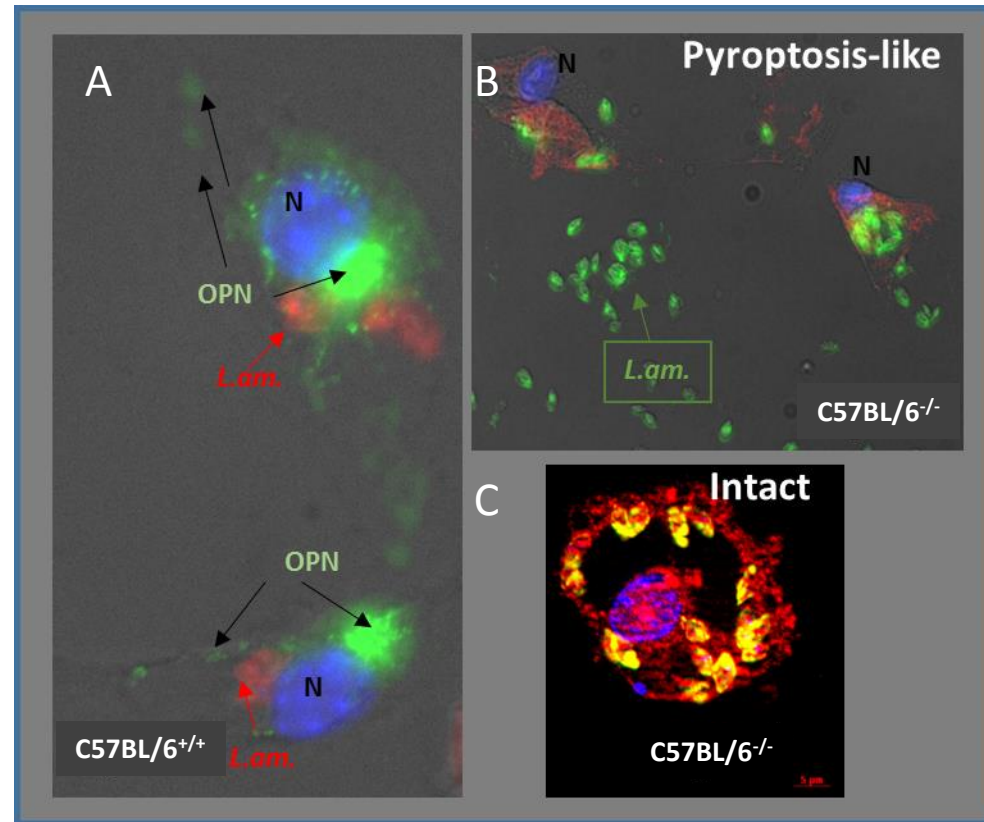

Supplement: Supplementary file 7 — Figure S5. BMF cell phenotypes in the presence and in the absence of osteopontin. The pyroptosis-like phenotype is observed in the absence of osteopontin indicating the implication of this protein in the cell adaptation to parasites. Immunostaining procedures are described in the Methods section. N: nuclei, 100x magnification. (PDF 215 kb) [file 12866_2019_1404_MOESM7_ESM.pdf]

**A**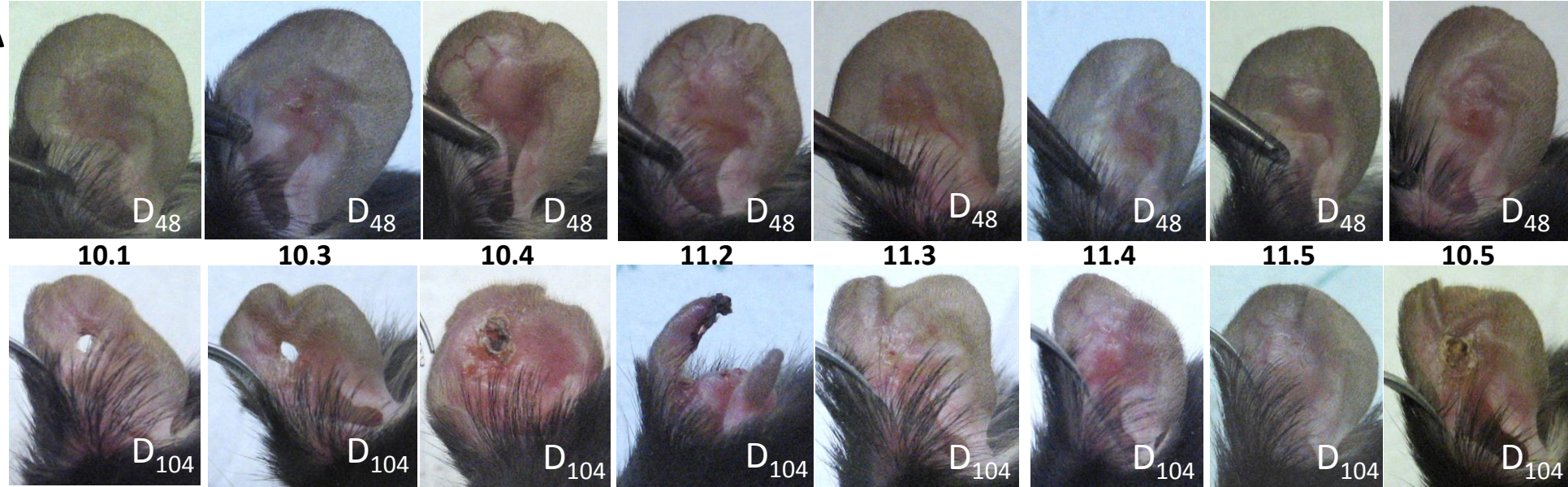**C57Bl/6<sup>+/+</sup>****B**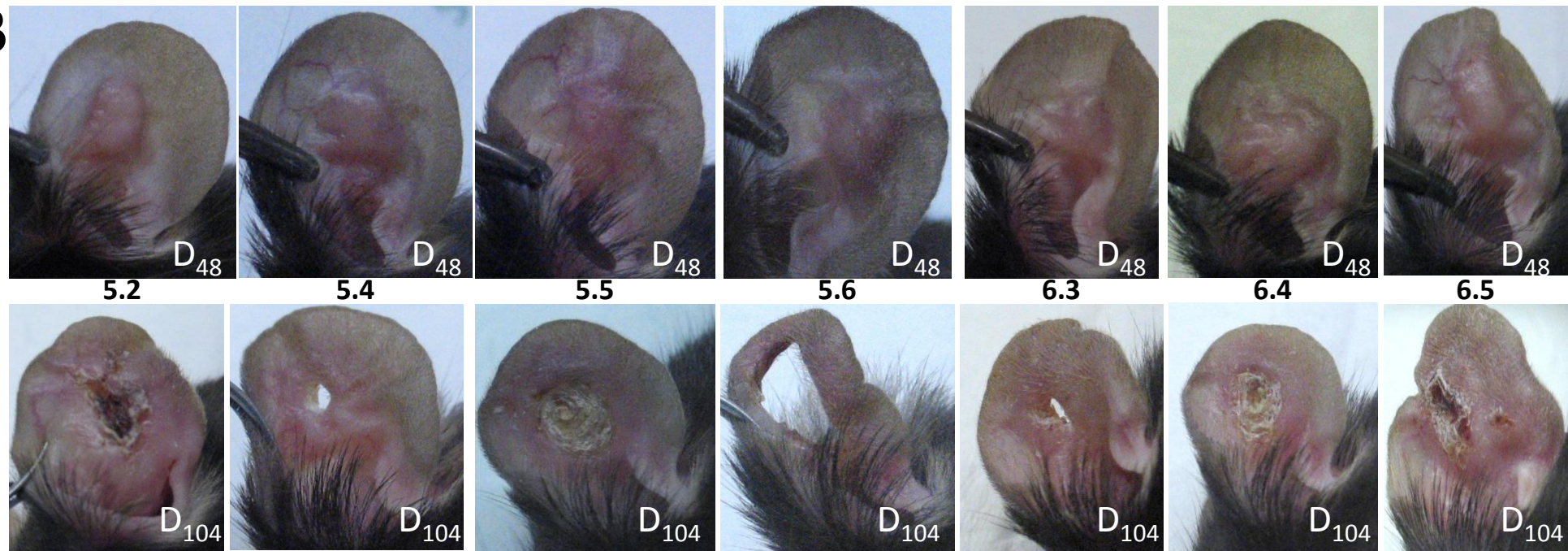**C57Bl/6<sup>-/-</sup>**

Supplement: Supplementary file 8 — Figure S6. Evolution of representative ear phenotypic lesions at Day 48 and 104. A. C57BL/6 wild type phenotypic scores at Day 48 p. i.: 8/10 (80%) mild inflammation. At Day 104: 4/10 (40%) moderate to severe inflammation and 6/10 (60%) tissue disrupted at the level of infection. B. C57BL/6−/− mice phenotypic scores at D48: 11/11 (100%) severe inflammatory lesions. At Day104: 7/11 (64%) destructive inflammatory lesions and 4/11 (36%) damaged tissue. (PDF 611 kb) [file 12866_2019_1404_MOESM8_ESM.pdf]

### A. Tissue Inflammation

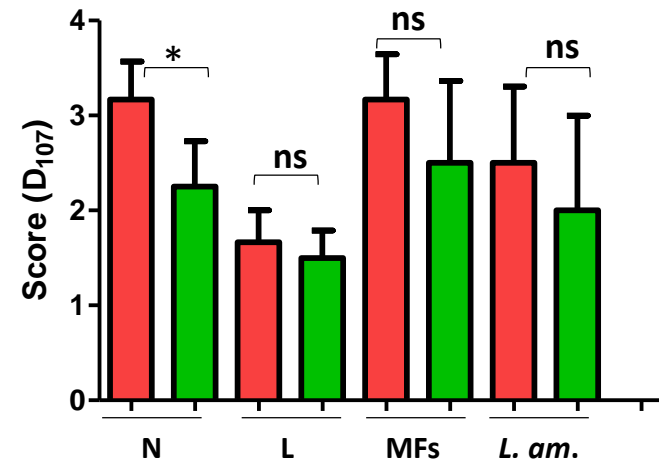

### B. Tissue destruction

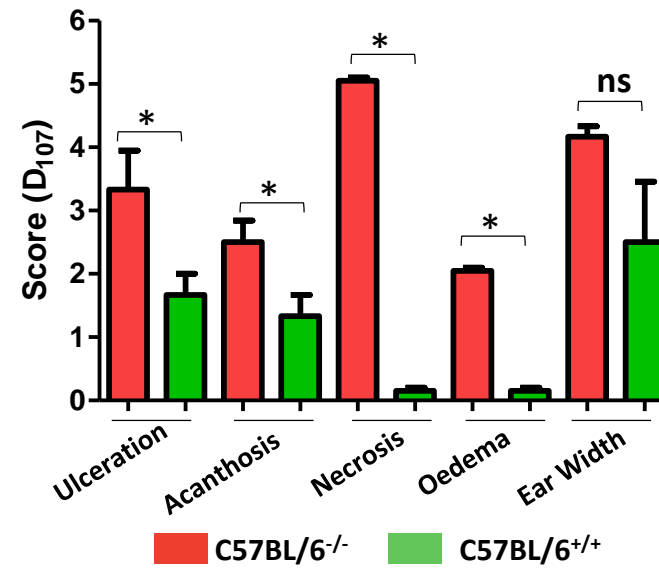

Supplement: Supplementary file 9 — Figure S7. Mean Histopathology scores. Mean values are from C57BL/6 wild type (green bars) and C57BL/6−/− (red bars) mice, post inoculated with 104 L. amazonensis metacyclic promastigotes. A. Tissue inflammation: N = Neutrophils; L = Lymphocytes, MFs = Macrophages. B. Tissue destruction. Number of mice studied: 6 KO; 4 WT. Unpaired t test with Welch’s correction, one tailed P-values. (PDF 270 kb) [file 12866_2019_1404_MOESM9_ESM.pdf]

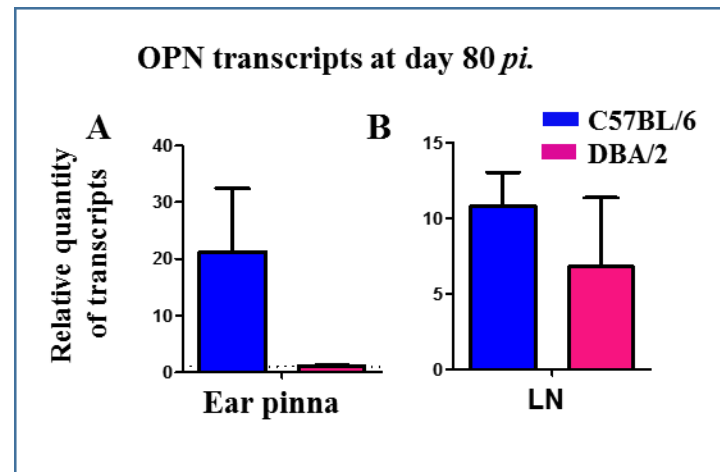

Supplement: Supplementary file 10 — Figure S8. Opn gene expression in C57BL/6 and DBA/2 mice in vivo. Real-time qPCR of opn transcripts in C57BL/6 (blue bars) and DBA/2 mice (red bars) at day 80 p.i. corresponding to the pic of opn transcripts observed (A) in the ear pinna (see Fig. 7a) and (B) in draining lymph nodes. (PDF 16 kb) [file 12866_2019_1404_MOESM10_ESM.pdf]
