# Supplementary material for: Osteopontin in the host response to Leishmania amazonensis
Source: BMC Microbiol. 2019 Feb 8;19:32. doi: 10.1186/s12866-019-1404-z (PMC6368773; doi:10.1186/s12866-019-1404-z)
Supplement: Supplementary file 2 — Table S1. Comparative statistical evaluation of parasite infectivity in BMF. Data are analysed by the Quantitative Parasitology software (QP3.0) [2] and are illustrated on Fig. 1 in the text. (PDF 13 kb) [file 12866_2019_1404_MOESM2_ESM.pdf]

**Table S1**: **Comparative statistical evaluation of parasite infectivity in BMF**.

**A. Parasitic infection**

| **Host (BMF)** | **Mean Intensity** | | **Mean Crowding** | | **Host size** | |
|---|---|---|---|---|---|---|
| | **24 h *pi*** | **48 h *pi*** | **24 h *pi*** | **48 h *pi*** | **24 h *pi*** | **48 h *pi*** |
| **C57BL/6$^{+/+}$ N° (SD)** | 2.88 (1.7) | 6.163 (5.064) | 3.84 | 10.28 | 68 | 141 |
| **C57BL/6$^{-/-}$ N° (SD)** | 3.63 (2.7) | 15.221 (11.1) | 5.68 | 23.28 | 46 | 86 |

**B. Parasitic load statistics**

| **Hosts** | **Prevalence (p-value)** | **Mean Intensity (p-value)** | **Mean Crowding** |
|---|---|---|---|
| **C57BL/6$^{+/+}$ 24h *vs* 48h** | 0.353 vs 0.695 (p=0.0001) | 0.00001 | P<0.05, CI 97.5% |
| **C57BL/6$^{-/-}$ 24h *vs* 48h** | 0.652 vs 0.925 (p=0.0001) | 0.00001 | P<0.05, CI 97.5% |
| **C57BL/6$^{+/+}$ 24h *vs* C57BL/6$^{-/-}$ 24h** | 0.353 vs 0.652 (p=0.002) | 0.21 NS | P>0.05 NS |
| **C57BL/6$^{+/+}$ 48h *vs* C57BL/6$^{-/-}$ 48h** | 0.695 vs 0.925 (p=0.0001) | 0.0001 | P<0.05, CI 97.5% |
