# Supplementary material for: Osteopontin in the host response to Leishmania amazonensis
Source: BMC Microbiol. 2019 Feb 8;19:32. doi: 10.1186/s12866-019-1404-z (PMC6368773; doi:10.1186/s12866-019-1404-z)
Supplement: Supplementary file 5 — Table S2. In situ detection scores of infected (INF), Non-Infected (Non-INF) and Pyroptosis-like (INF-PYR) BMFs. Numbers of cells correspond to counts of at least 4 different image fields under grilles of immunofluorescence slides (40x magnification) using epifluorescence microscopy. Parasites, vacuoles and nuclei were labelled and evaluated using ApoTome and MosaiX software as described in the Methods section. Data are presented as graph on Fig. 3a. (PDF 166 kb) [file 12866_2019_1404_MOESM5_ESM.pdf]

Table S2. *In situ* detection scores of infected (INF), Non-Infected (Non-INF) and Pyroptosis-like (INF-PYR) BMFs. Numbers of cells correspond to counts of at least 4 different image fields under grilles of immunofluorescence slides (40x magnification) using epifluorescence microscopy. Parasites, vacuoles and nuclei were labelled and evaluated using ApoTome and MosaiX software as described in the Methods section. Data are presented as graph in the text on Fig. 3A.

**Cell Phenotype**	**C57BL/6$^{+/+}$**		**C57BL/6$^{-/-}$**	
	**24H *p.i. (%)***	**48H *p.i. (%)***	**24H *.p.i (%)***	**48H *p.i. (%)***
**INF**	74 *(36%)*	128 *(61%)*	243 *(76.4%)*	29 *(43.28%)*
**INF-PYR**	0	0	45 *(14.15%)*	32 *(47.76%)*
**Non-INF**	132 *(64%)*	82 *(39%)*	30 *(9.43%)*	6 *(8.9%)*
**TOTAL**	**206**	**210**	**318**	**67**
